# The evolving mpox threat (2022-2024): clade dynamics, immune evasion, and escalating global health challenges

**DOI:** 10.3389/fcimb.2025.1677762

**Published:** 2026-02-09

**Authors:** Muhammad Irfan Khan, Ahmed A. Saleh, Rahmat Ali, Rifat Ullah Jan, Jie Gu, Ji Dejun

**Affiliations:** 1 College of Animal Sciences and Technology, Yangzhou University, Yangzhou, Jiangsu, China; 2 Animal and Fish Production Department, Faculty of Agriculture (Al-Shatby), Alexandria University, Alexandria, Egypt

**Keywords:** diagnostics, epidemiology, immune evasion, mpox, reproductive health, sexual transmission, transmission, vaccination

## Abstract

The 2022–2024 global Mpox outbreak, declared a Public Health Emergency of International Concern, marks a pivotal shift in the virus’s epidemiology, extending beyond its traditional endemic regions in Africa. This review provides a comprehensive synthesis of the evolving Mpox threat, analyzing the dynamics of MPXV clades (I, Ia/Ib, II, IIa/IIb), with a focus on the enhanced transmissibility of the emergent Clade Ib variant linked to APOBEC3-mediated mutations. We detail the virus’s genetic and structural characteristics, its unique cytoplasmic replication cycle, and sophisticated immune evasion strategies, including the interference with type I interferon signaling and modulation of pro-inflammatory cytokines. The review examines changing transmission paradigms, highlighting the role of sustained human-to-human and sexual transmission in recent outbreaks, and discusses the clinical spectrum of disease, from classic febrile rash to atypical presentations and severe outcomes in immunocompromised individuals. A significant portion of this analysis is dedicated to the profound implications for reproductive health, covering vertical transmission with high rates of fetal loss, viral persistence in semen, and potential impacts on fertility. We evaluate current diagnostic standards, such as PCR, alongside emerging techniques, and assess the efficacy of antiviral therapies, including tecovirimat, brincidofovir, and cidofovir. The status of vaccination, from second-generation (ACAM2000) to third-generation (MVA-BN and LC16m8) platforms, is critically appraised for its role in outbreak control. Furthermore, we explore the successes and hurdles of public health strategies, including surveillance, contact tracing, and community engagement, in managing stigma and ensuring equity. The review concludes by outlining future perspectives, emphasizing the urgent need for enhanced surveillance, accessible countermeasures, and research into broad-spectrum antivirals and vaccines to prepare for the ongoing threat of MPXV.

## 1 Background

In the aftermath of the unprecedented global crisis precipitated by the COVID-19 pandemic, which significantly influenced societal structures and economic systems across the globe, the international community currently experiences heightened vulnerability, exhibiting an augmented predisposition to the advent of an alternative viral pathogen scientifically designated as “Monkeypox.” Mpox is a zoonotic viral disease caused by the Mpox virus (MPXV). MPXV is a double-stranded DNA pathogen classified within the *Orthopoxvirus* genus of the *Poxviridae* family, sharing lineage with smallpox and vaccinia (Di Giulio and Eckburg, 2004; Magnus et al., 1959). The virus transmits from animal reservoirs to humans (primary zoonosis) and can also spread between humans, posing significant public health risks (Bunge et al., 2022). The disease was originally named ‘monkeypox’ following its first detection in laboratory monkeys in Denmark in 1959 (Magnus et al., 1959). In a significant nomenclature shift aimed at reducing stigma, the World Health Organization (WHO) proposed on November 28, 2022, that the term ‘Mpox’ be adopted as the preferred synonym. A one-year transition period was recommended, during which both names would coexist, followed by the phasing out of the original term (WHO, 2022a Mpox Outbreak: Global Trends, 2023). Human infection was first documented in 1970 in Zaire (DRC) in an infant (Breman et al., 1980). This marked the beginning of the virus’s known impact on human populations, leading to its endemic establishment in Central and West Africa. This review provides a chronological overview of these and subsequent key milestones in the history of Mpox. It was not until 2003 that cases appeared beyond the African continent (CDC, 2003b). MPXV comprises two primary clades with distinct subclades per WHO classification: a) Clade I(subclades Ia/Ib), historically termed ‘Congo Basin,’ is endemic to Central Africa and associated with higher virulence (historical fatality: ≤10%, recent: 1–3.3%). b) Clade II (subclades IIa/IIb), previously ‘West African,’ is endemic to West Africa and less virulent (survival >99.9%). Clade IIb caused the 2022 global outbreak (CDC, 2022). The most common MPXV symptoms include rash (Hammad et al., 2024; Isaacs, 2022), fever (Dhana et al., 2022; Mukareem Ali et al., 2025; Saraswat and Shah, 2024), backaches (Buerke et al., 2025), shivering (Gupta et al., 2023; Lucena-Neto et al., 2023), muscular pain (Singh et al., 2024), lymphadenopathy (Ci Ng et al., 2023; Zahmatyar et al., 2023), and headache (Mukareem Ali et al., 2025; Xie et al., 2022); however, the presence of swollen lymph nodes at the outset of fever helps to distinguish MPXV from smallpox, although both diseases can also be asymptomatic. The immunological cross-reactivity between Mpox and smallpox permits the strategic deployment of smallpox vaccines according to WHO guidelines (Smallpox vaccines, 2024). Current medical countermeasures in endemic zones incorporate these vaccines alongside specific antivirals like tecovirimat (Albin et al., 2022; Beer and Rao, 2019; Ilic et al., 2022; Selb et al., 2022). The new Clade I variant exhibits increased transmissibility compared to previous Clade I strains, while its fatality rate remains comparable, which is a critical point. Earlier Clade I strains are known to be more fatal than Clade IIb (Verma et al., 2024). For instance, mpox infections among pregnant women caused by Clade I were reported to have a 75% perinatal case fatality rate in the DRC (Masirika et al., 2024). The enhanced transmissibility of these new Clade I variants has been noted in several studies. For example, the Clade Ib variant is characterized by enhanced transmissibility, particularly through human-to-human contact, including sexual transmission (Vakaniaki et al., 2024). This enhanced human-to-human transmission is a significant concern for public health, as it increases the potential for wider spread and larger outbreaks (Lourie et al., 1972). The 2024 mpox outbreak in the DRC experienced a rapid surge in cases, characterized by sustained human-to-human transmission of a new Clade I lineage (Kinganda-Lusamaki et al., 2024; Wawina-Bokalanga et al., 2024).

While the fatality rate of these newer Clade I variants is stated to be comparable to previous Clade I strains, it is essential to remember that Clade I is inherently more severe than Clade II (Butler and Banday, 2023; Greenwald et al., 2023). The high mortality associated with Clade I mpox, particularly in vulnerable populations and regions with limited healthcare access, remains a serious issue (Srivastava et al., 2024). The potential for widespread transmission of a strain that maintains a high fatality rate could lead to a more devastating public health crisis than observed with the 2022 global outbreak. The ability of Clade I to spread through sexual contact, a mode of transmission predominantly observed with Clade IIb, further exacerbates the risk.

## 2 MPXV: genetic and structural features

MPXV is one of the viruses belonging to the *Orthopoxvirus* genus, which is classified under the *Poxviridae* family (Reda et al., 2023). MPXV is an extensive double-stranded DNA genome roughly 197 kb long and encodes 209 predicted open reading frames (Choi et al., 2022). The virus particle’s rectangular shape is enveloped by a membrane composed of lipids and proteins that contains various viral proteins, such as hemagglutinin, envelope antigens, and A-type inclusion bodies (Lansiaux et al., 2022). *Orthopoxviruses* are characterized by their conserved central core genes, which are essential for replication and structure, while their terminal ends exhibit variability that influences host range, virulence, and immunogenicity. MPXV can be classified into two primary clades: clade I and clade II. The Congo Basin clade is associated with higher rates of illness and death (Kindrachuk et al., 2012).

### 2.1 Biological features of MPXV

**Figure 1:**
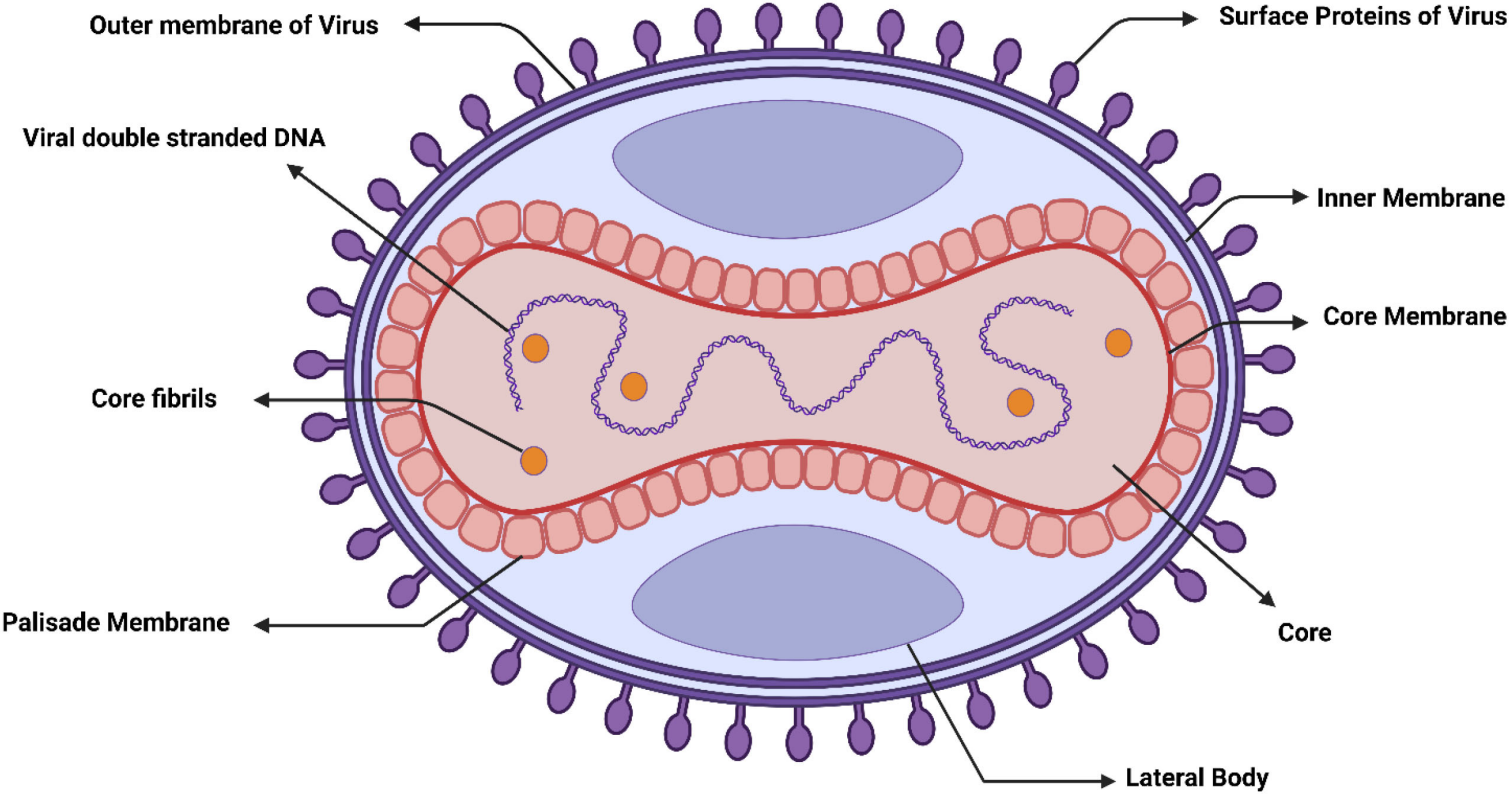
Schematic structure of the Monkeypox virus (MPXV) particle. The virion showcases the central core containing the double-stranded DNA genome, flanked by lateral bodies, and enclosed by the core membrane and palisade layer. The inner membrane surrounds the core structure. The outer envelope, derived from the host cell, is studded with viral proteins critical for attachment and entry into host cells. Key proteins discussed in the text are indicated, including surface proteins involved in cell adhesion and the F13 protein (VP37), located in the outer envelope, which is the molecular target of the antiviral drug Tecovirimat. (Created with BioRender.com).

The MPXV virion is a substantial, brick-shaped particle approximately 200–250 nm in length. Its complex, multi-layered architecture is key to its infectivity. At the center lies the viral core, a barbell-shaped nucleoprotein complex housing the ~197 kb double-stranded DNA genome. This core is encased by the core membrane and a surrounding proteinaceous palisade layer, which together provide structural integrity. The entire core structure is bound by an inner membrane. Flanking the core are two lateral bodies; their precise function remains unclear but may involve virion assembly and egress. During budding from the host cell, the particle acquires an additional outer envelope derived from the host membrane. Studded with viral proteins such as the entry-fusion complex and adhesion molecules, this outer envelope is essential for attachment to and entry into new host cells (Martínez-Fernández et al., 2023; Rampogu et al., 2023; Kataria et al., 2023; Li et al., 2024). The complete structure is illustrated in **Figure 1**.

The phenomenon of viral infection initiates with the virus’s adhesion to specific receptors, encompassing glycosaminoglycans, followed by the fusion of viral and cellular membranes. Viral replication occurs solely in the cytoplasm, yielding two infectious agents: intracellular mature virions (IMV) and extracellular enveloped virions (EEV). These forms are imperative for the propagation of the virus, both within a singular host and among diverse hosts (Yadav et al., 2022). MPXV utilizes host cellular mechanisms for gene expression, DNA replication, and particle assembly. Key virulence factors encompass immune evasion strategies encoded by specific genes, including K3L, E3L, and C7L, as well as cytokine response modifiers. From a diagnostic perspective, MPXV generates distinctive *Orthopoxvirus* cytoplasmic inclusions and virion aggregations, which are discernible through microscopic analysis. Confirmation of MPXV infection is achieved through serological and nucleic acid detection techniques, while virus isolation requires cultivation in laboratory settings. Genomic sequencing provides additional insights into viral lineage and genetic variations. Animal models are valuable tools for investigating pathogenesis and assessing potential medical interventions (Guarner et al., 2004).

### 2.2 Mpox virus immune evasion strategies

MPXV, in common with other orthopoxviruses, has evolved sophisticated mechanisms to counteract the host’s innate immune system, a cornerstone of its pathogenicity (Jia, 2024; Pashazadeh Azari et al., 2024; Zhu et al., 2023). A primary target for this evasion is the type I interferon (IFN) response, a critical component of the innate antiviral defense (Kikkert, 2020; Lum and Cristea, 2022; Manfrini et al., 2024). The virus employs a multi-layered strategy, broadly conserved within the Orthopoxvirus genus, to inhibit both the production of IFNs and the signaling cascades of already secreted IFN (Saghazadeh and Rezaei, 2023). MPXV encodes homologs of vaccinia virus (VACV) immunomodulatory proteins that disrupt IFN signaling. A key example is the F3L protein, a homolog of VACV E3, which is crucial for blocking activation of the cellular innate immune system (Arndt et al., 2015). Although the MPXV F3L gene is predicted to encode a protein with an N-terminal truncation compared to VACV E3, its role in immune evasion is significant.

### 2.3 Specific evasion mechanisms employed by MPXV

#### 2.3.1 Interference with IFN signaling

MPXV, like other poxviruses, employs various strategies to counteract type I IFN production and signaling (Saghazadeh and Rezaei, 2023). This includes blocking the sensing of viral components by pattern recognition receptors (PRRs) and inhibiting downstream signaling pathways (Kasuga et al., 2021; Manfrini et al., 2024; Murray et al., 2018). Furthermore, MPXV has been shown to directly impair IFN expression and actively interfere with the IFN/ISG axis (Bordi et al., 2024).

#### 2.3.2 Role of APOBEC3 mutations in viral evolution

In addition to the structural and functional characteristics of the MPXV particle, recent research has shed light on the role of APOBEC3 mutations in the newly identified Clade I variant, which is a critical aspect. APOBEC3 (Apolipoprotein B mRNA Editing Catalytic Polypeptide-like 3) proteins are a family of host cytidine deaminases that play a crucial role in innate immunity against viruses (Jakobsdottir et al., 2022; Schwartz and Pittman, 2023). These enzymes induce mutations by deaminating cytosine to uracil in viral DNA during replication, resulting in the hypermutation of the viral genome (Chen et al., 2021; Probert et al., 2025). This process can significantly reduce viral fitness and, in many cases, inactivate the virus. However, the interplay between APOBEC3 and viral evolution is a complex phenomenon. While APOBEC3 can act as an antiviral defense, some viruses, including MPXV, can adapt to or even exploit APOBEC3 activity.

While APOBEC3 can act as an antiviral defense, some viruses, including MPXV, can adapt to or even exploit APOBEC3 activity (Probert et al., 2025). Recent genomic analyses have shown an enrichment of APOBEC3-signature mutations (G-to-A and C-to-T transitions in specific trinucleotide contexts) in circulating MPXV strains, particularly the emerging Clade Ib lineage (Kinganda-Lusamaki et al., 2024; Wawina-Bokalanga et al., 2024). The critical question is whether these mutations are merely bystander effects or are being selected for because they confer a fitness advantage. The localization of these mutations in specific genes provides compelling, though not yet definitive, hypotheses for a causal link to enhanced transmission.

### 2.4 Link between APOBEC3 mutations and clade Ib spread

The recent emergence of Clade Ib MPXV, particularly in the Democratic Republic of Congo (DRC), has been associated with sustained human-to-human transmission (Kinganda-Lusamaki et al., 2024; Vakaniaki et al., 2024 A; Wawina-Bokalanga et al., 2024). Several studies suggest that APOBEC3-mediated mutagenesis contributes to the rapid spread and distinct characteristics of this new subclade (Kinganda-Lusamaki et al., 2024), generated high-quality genomes from 337 patients in the DRC and found an “enrichment of APOBEC3 mutations linked to human adaptation.” This suggests that APOBEC3 activity is not merely an antiviral defense but also a driver of viral evolution that can enhance human-to-human transmission.

The sustained human-to-human transmission of Clade Ib MPXV is strongly linked to its ongoing microevolution, which appears to be accelerated by host-driven mutagenesis. Genomic studies confirm that APOBEC3-induced mutations, primarily G-to-A transitions, are introducing nonsynonymous amino acid changes across the viral genome (Alakunle et al., 2024; Deiana et al., 2024). The central hypothesis is that a subset of these mutations is being selectively enriched because they confer a fitness advantage in human hosts, moving beyond correlation to a plausible causal mechanism for enhanced transmission.

Several key viral proteins are hypothesized to be functionally altered by these mutations. In immune evasion pathways, genes such as E6R (a poxviral Golgi anti-apoptotic protein) and A46R (a modulator of TLR signaling) have been identified as mutation targets. Alterations here could enhance the virus’s ability to suppress early innate immune detection in human cells, facilitating more efficient initial replication. Furthermore, mutations have been documented in genes encoding the entry/fusion complex, including A44L (homolog of VACV H3) and D14L (homolog of VACV A27). Amino acid substitutions in these surface proteins could increase the virus’s affinity for human cellular receptors or alter its tropism for mucosal and keratinocyte cells, which are critical portals for sexual and close-contact transmission (Alakunle et al., 2024; Deiana et al., 2024).

The translation of these molecular changes into enhanced human-to-human transmission is theorized to operate through a multi-faceted mechanism. This includes: 1) Increased Viral Shedding from higher replication efficiency, leading to a greater infectious dose in bodily fluids; 2) Prolonged Infection duration due to more effective suppression of host apoptosis or interferon responses, extending the shedding window; and 3) Altered Cell Tropism that broadens the range of susceptible human cell types. While these links are strongly suggested by genomic epidemiology, it is crucial to state that direct functional validation through reverse genetics and *in vitro* models is required to confirm causality. The current evidence robustly positions APOBEC3 activity as a key driver of MPXV’s adaptive evolution, with specific mutations in immune and structural genes being prime candidates for the heightened transmissibility observed in the Clade Ib outbreak.

### 2.5 Modulation of pro-inflammatory cytokines

MPXV’s interference with the host immune response extends beyond the IFN system to a broader modulation of pro-inflammatory cytokines and chemokines, which are crucial for orchestrating immune cell recruitment and function. This is achieved through an arsenal of viral immunomodulatory proteins, many of which are homologs of well-characterized vaccinia virus (VACV) proteins. The virus employs strategies including the secretion of decoy receptors and proteins that directly block intracellular signaling pathways (Dong et al., 2025).

For instance, MPXV encodes a homolog of the VACV cytokine response modifier (Crm) proteins. While direct characterization in MPXV is ongoing, VACV CrmB and CrmD are known to bind and inhibit TNF-α, a master regulator of inflammation. Similarly, a homolog of the VACV IL-1β receptor (B16R) is predicted to sequester IL-1β, preventing its pro-inflammatory signaling. Furthermore, the F3L protein, in addition to its role in IFN evasion, is implicated in broadly suppressing the nuclear factor kappa B (NF-κB) signaling pathway, which is central to the production of cytokines like IL-6, IL-1β, and TNF-α (Arndt et al., 2015). The recently characterized PoxS protein acts by sequestering STAT2, thereby disrupting signaling not only for IFNs but also potentially dampening the inflammatory responses they help to initiate (Chan et al., 2025). A summary of these key immunomodulatory proteins and their targets is provided in **Table 1**.

**Table 1:** Key MPXV immunomodulatory proteins targeting cytokine and chemokine responses.

MPXV protein (VACV homolog)	Target/mechanism	Functional consequence
F3L (E3L)	Inhibits PKR activation; suppresses NF-κB signaling.	Broadly reduces production of pro-inflammatory cytokines (e.g., IL-6, IL-1β, TNF-α).
PoxS	Sequesters STAT2.	Disrupts IFN signaling and associated inflammatory gene activation.
CrmB/CrmD homolog	Soluble TNF Receptor; binds and neutralizes TNF-α.	Inhibits TNF-α-mediated inflammation and cell death.
B16R homolog	Soluble IL-1β Receptor; binds and neutralizes IL-1β.	Blocks IL-1β pro-inflammatory signaling.
vCCI/CBP II homolog	Binds a broad range of CC-chemokines (e.g., MIP-1α, RANTES).	Prevents chemokine interaction with host receptors, impairing leukocyte recruitment.

An excessive immune response can lead to a “cytokine storm,” as seen in conditions like sepsis and severe COVID-19, where dysregulated cytokine production causes hyperinflammation and organ failure (Hobbs et al., 2024; Mińko et al., 2024). Conversely, a dampened inflammatory response, as induced by MPXV, can allow the virus to escape immune detection and clearance.

### 2.6 Impact on immune cell recruitment and function

Chemokines are crucial for recruiting immune cells, such as T cells, neutrophils, dendritic cells, and macrophages, to sites of infection (Karimi et al., 2024). By interfering with chemokine production, MPXV can hinder the timely arrival of these essential immune cells, thereby delaying or preventing an effective antiviral response. This dampening of the inflammatory response helps the virus avoid immune surveillance and establish persistent infection (Bordi et al., 2024). The overall picture of MPXV infection demonstrates a complex interplay with the host immune system. MPXV can infect keratinocytes, fibroblasts, and dendritic cells, leading to local immune activation and subsequent systemic spread via lymphatic vessels and viremia (Singh et al., 2024).

### 2.7 MPXV infection and neutralizing antibody response:

**Figure 2:**
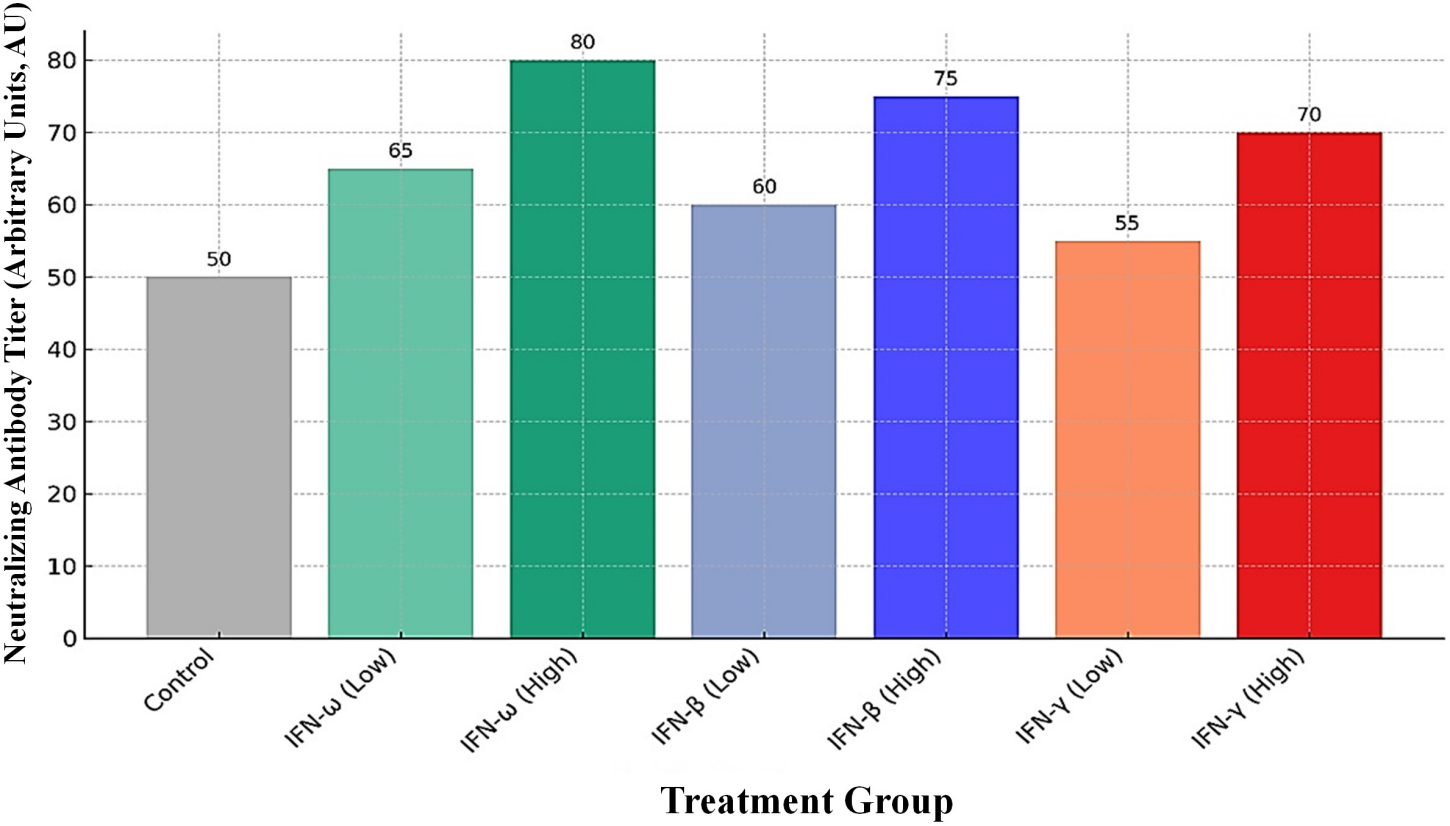
Modulatory effects of IFN-ω, IFN-β, and IFN-γ on MPXV neutralizing antibody titers in a dose-dependent and immunotype-specific manner.

MPXV infection triggers a significant immune response in the host, particularly the production of neutralizing antibodies, which are critical for protection. Recent research highlights notable differences in the quality and durability of this antibody response when induced by natural MPXV infection compared to Modified Vaccinia Ankara-Bavarian Nordic (MVA-BN) vaccination (Selverian et al., 2025). The impact of different interferons on MPXV neutralizing antibody titers has been investigated. For example, IFN-ω, IFN-β, and IFN-γ were found to modulate these titers in a concentration-dependent manner, with notable differences observed in antibody responses compared to the control groups. The impact of different interferons on MPXV neutralizing antibody titers has been investigated. For example, IFN-ω, IFN-β, and IFN-γ were found to modulate these titers in a concentration-dependent manner, with notable differences observed in antibody responses compared to the control groups. Evidence from existing studies suggests that, despite MPXV’s known strategies to evade interferon-mediated responses, interferons may still play a modulatory role in shaping the host’s antibody response. As shown in **Figure 2**, the modulatory effects of IFN-ω, IFN-β, and IFN-γ on MPXV neutralizing antibody titers are both dose-dependent and immunotype-specific, highlighting the complex interplay between the virus and the host’s humoral immunity.

### 2.8 Interferon-stimulated genes and MPXV

**Figure 3:**
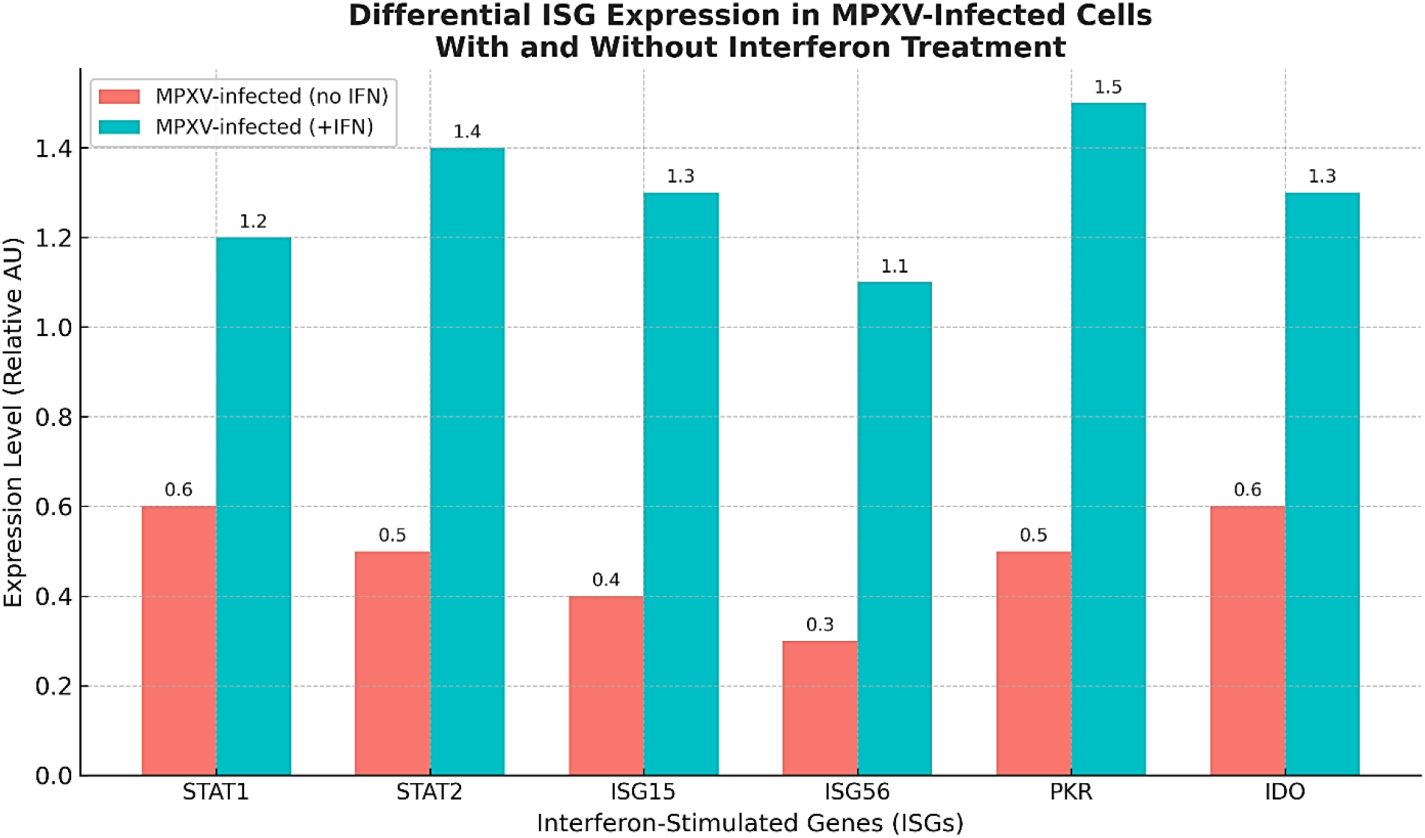
Expression levels of key ISGs (STAT1, STAT2, ISG15, ISG56, PKR, IDO) in MPXV-infected cells with and without interferon treatment. IFN treatment increases ISG expression despite MPXV immune evasion. Values are shown as normalized arbitrary units (AU).

Studies in MPXV-infected cells have demonstrated altered expression of key interferon-stimulated genes (ISGs), including STAT1, STAT2, ISG15, ISG56, PKR, and IDO (**Figure 3**), which are crucial for establishing an antiviral state (Bordi et al., 2024; Jiang et al., 2024). This active interference with the IFN/ISG axis is a critical feature of MPXV’s strategy to dismantle the host’s antiviral state. Notably, type I and II interferons still exert measurable anti-MPXV effects, underscoring a host-pathogen battle where the host’s antiviral attempts persist despite viral evasion (Bordi et al., 2024 & Jiang et al., 2024; Rowe et al., 2024).

**Figure 3** visually summarizes the expression levels of key ISGs (STAT1, STAT2, ISG15, ISG56, PKR, IDO) in MPXV-infected cells with and without interferon treatment. The data show that IFN treatment increases ISG expression despite MPXV’s immune evasion efforts, demonstrating the host’s ongoing attempt to mount an effective antiviral state against the infection.

ISGs play diverse roles in mounting an antiviral state. While some ISGs have specific,well-characterized antiviral functions, many others are still being elucidated (Schoggins and Rice, 2011). Studies have shown that ISG15 knockout in mice impairs IFNα-mediated antiviral activity and increases susceptibility to viral infections, such as Pseudorabies virus (PRV) (Holthaus et al., 2019; Li et al., 2022; Liu et al., 2022). ISG15 has also been implicated in controlling Dengue and Zika virus replication through stabilizing its binding partner USP18, which competes with viral proteins for STAT2 degradation (Espada et al., 2024).

The ongoing battle between MPXV and the host IFN-ISG system has significant clinical implications. The ability of MPXV to evade immune responses contributes to its pathogenicity and spread (Alakunle et al., 2024). However, the observed partial sensitivity of MPXV to IFN-γ antiviral effects suggests therapeutic potential for IFN-based treatments. Indeed, IFN-I has been shown to reduce Mpox pathogenicity in animal models, highlighting its potential as a therapeutic intervention (Zhu et al., 2025). This aligns with broader research indicating the therapeutic utility of interferons against various viral infections, including COVID-19 (Altmann, 2023; Lin and Shen, 2020).

Further research is needed to fully characterize the specific ISGs most critical for controlling MPXV infection and to understand the precise viral mechanisms that counteract each of them. This knowledge could lead to the development of novel host-directed therapies that bolster the innate immune response against MPXV, potentially by enhancing ISG expression or function, or by counteracting specific viral immune evasion proteins. Understanding the host-pathogen interplay at this molecular level is critical for future therapeutic interventions and pandemic preparedness.

## 3 The life cycle of MPXV

The MPXV lifecycle follows a complex, multistage process, beginning with host cell entry and culminating in the release of mature virions, which enable subsequent rounds of infection (Letafati and Sakhavarz, 2023). Unlike most DNA viruses, MPXV uniquely replicates exclusively within the host cytoplasm (Buller and Palumbo, 1991).

### 3.1 Host cell entry

Upon entering a host cell, MPXV follows a replication cycle conserved among poxviruses, beginning with entry and culminating in the release of mature virions (Letafati and Sakhavarz, 2023). A hallmark of poxvirus biology is the production of two distinct infectious forms, IMV and EEV, which originate from infected host cells and utilize different entry pathways (Alakunle and Okeke, 2022; Smith et al., 2002). Broadly across poxviruses, IMVs are thought to trigger micropinocytosis, while EEVs engage in direct membrane fusion (Buller and Palumbo, 1991). The initial attachment is enhanced by interactions with various host glycosaminoglycans, and studies of the prototypical vaccinia virus have identified proteins such as A34R, A26L, A27L, D8L, and H3L as fundamental for association with the cell membrane (Berhanu et al., 2008; Davies et al., 2005; Lin et al., 2000; Matho et al., 2018; Thirunavukarasu et al., 2013). For MPXV, the core entry mechanism following attachment involves membrane fusion and core release into the cytosol (Moss, 2016). Based on high conservation with vaccinia virus, this critical penetration step is understood to require the coordinated action of a multi-protein fusion complex, with MPXV homologs of the VACV proteins A27, A28, L1, F9, H2, and L5 (Diesterbeck et al., 2018; Foo et al., 2009; Laliberte et al., 2011).

**Figure 4:**
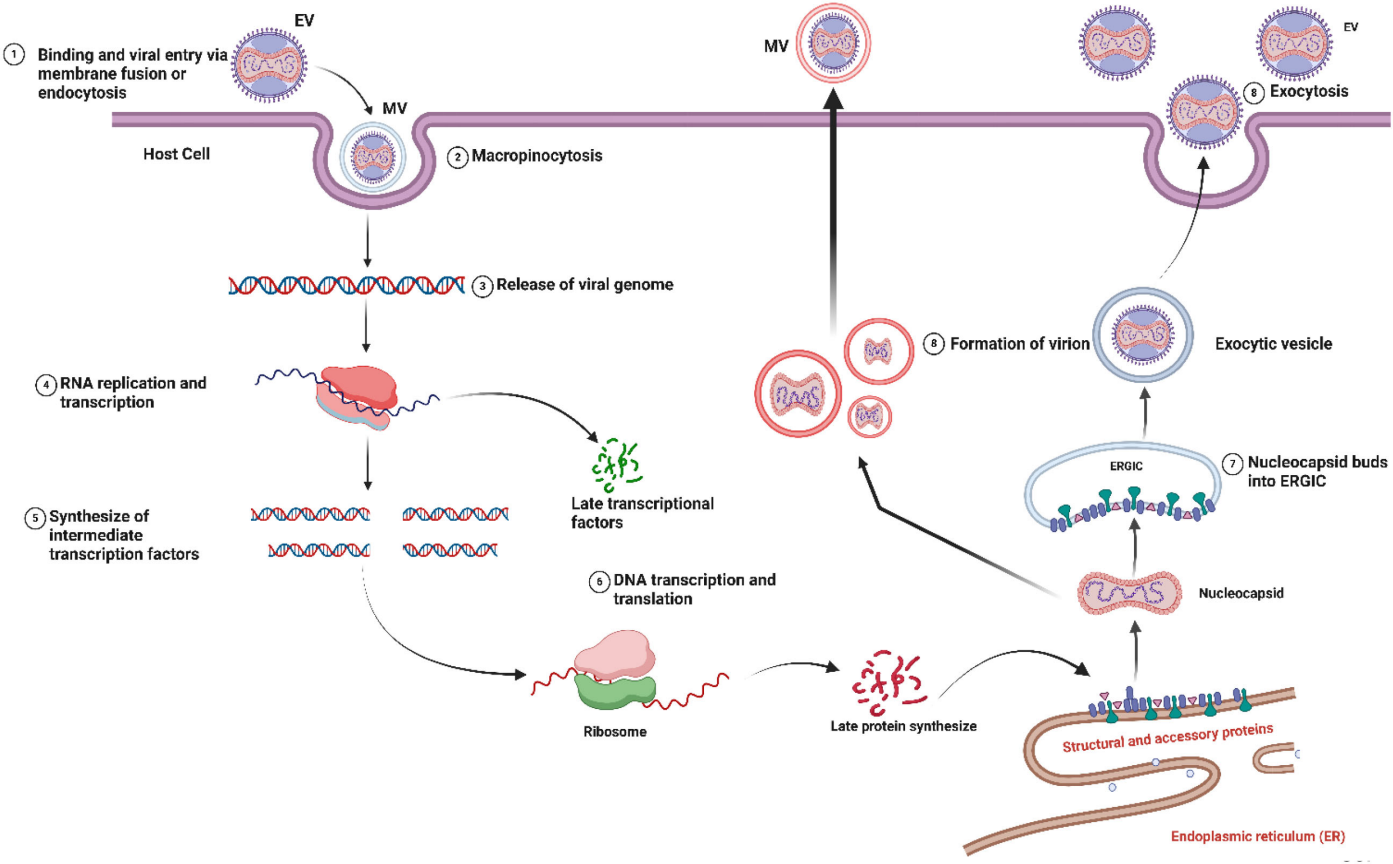
The replication cycle of Monkeypox virus (MPXV) within a host cell. The cycle begins with 1) viral attachment and entry via membrane fusion, mediated by the viral entry-fusion complex (includes proteins such as A27, L1, and H2). 2) The viral core is released into the cytoplasm, where early mRNA is transcribed and translated. 3) Genome replication occurs in cytoplasmic factories. 4) Intermediate and late gene expression leads to the synthesis of structural proteins and assembly of immature virions. 5) Virion maturation produces Intracellular Mature Virions (IMVs). 6) A subset of IMVs is wrapped by trans-Golgi or endosomal membranes to form Intracellular Enveloped Virions (IEVs). 7) IEVs are transported to the cell periphery. 8) The outer membrane fuses with the plasma membrane, releasing Extracellular Enveloped Virions (EEVs). The F13 protein plays a critical role in the wrapping process (step 6) and egress of enveloped virions, making it a key target for antiviral therapy. (Created with BioRender.com).

The delivered core packages early transcriptional machinery, while genome duplication is spatially segregated into distinct perinuclear replication factories (Faye et al., 2018). Genetic replication is catalyzed by a tripartite enzyme complex featuring the F8 polymerase, A22 processivity factor, and E4 glycosylase (Greseth and Traktman, 2022). The onset of viral transcription is mediated by a virally encoded, DNA-dependent RNA polymerase, a multi-subunit enzyme that directly transcribes the viral genome. The resulting mRNA transcripts are translated by host ribosomes, yielding temporally regulated viral proteins (early, intermediate, and late phases). Notably, while early transcription is driven solely by viral machinery, the expression of intermediate and late genes becomes dependent on host-derived transcription factors (McFadden, 2005; Wright et al., 1998). In the cytoplasm, the predominant form of the virus exists as IMVs, encased in a protein framework. According to (Roberts and Smith, 2008), certain virus particles may develop an extra coat, enabling them to cling to the target cell membrane as intracellular enveloped virions (IEVs). Both EEVs and cell surface-binding contained virions (CEVs) facilitate systemic and cell-to-cell transmission, respectively (**Figure 4**) (Pauli et al., 2010).

**Figure 4** provides a schematic overview of the MPXV replication cycle within a human host cell. The process begins with viral entry via membrane fusion or endocytosis, leading to the release of the viral core into the cytosol. This is followed by early gene expression, genome replication in cytoplasmic factories, and the subsequent assembly of new virions. **Figure 4** delineates the formation of both IMVs, which are released upon cell lysis, and EEVs, which are released via exocytosis and are critical for systemic spread.

### 3.2 Modes of transmission and epidemiological insights

The transmission of the Mpox virus primarily occurs via two main pathways: zoonotic transfer from animals to humans and subsequent human-to-human transmission. Zoonotic transmission takes place through direct exposure to the blood, bodily fluids, or lesion materials of infected animals. Although the natural animal reservoir has not yet been conclusively identified, African rodents and nonhuman primates are potential hosts and vectors facilitating human infection (Khattak et al., 2023). According to (Huston et al., 2023), Mpox is fundamentally understood as a zoonotic disease, with a transmission route involving animals, especially rodents, to human hosts. For example, primary animals include rope squirrels, tree squirrels, Gambian pouched rats, and dormice. This transmission pathway remains significant in endemic territories where interactions between humans and infected animal species are more frequent. Another finding has also demonstrated that the virus can be transmitted via urinary and fecal matter, suggesting an alternative pathway for infection (Huston et al., 2023).

**Figure 5:**
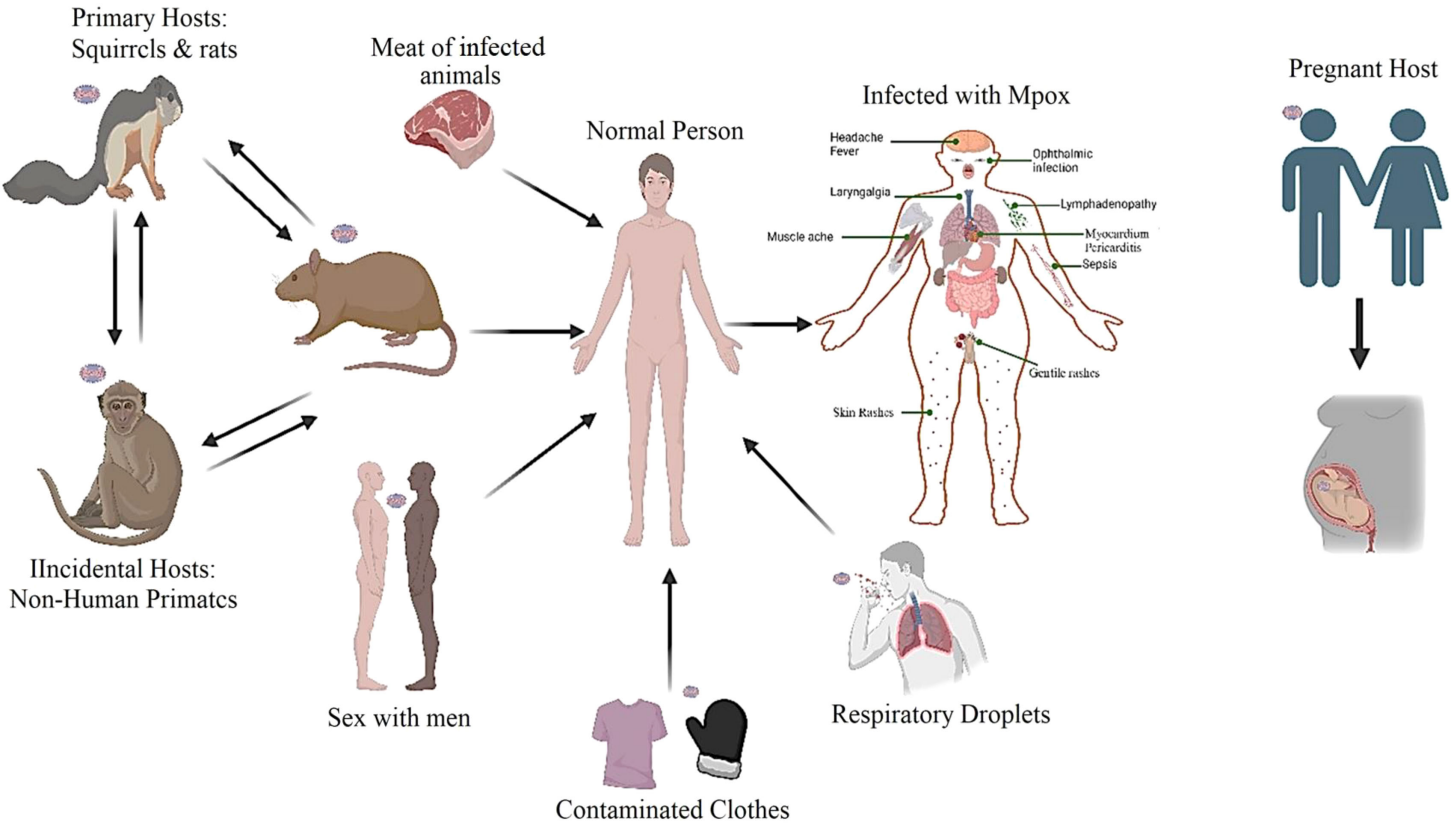
The diverse transmission mechanisms of MPXV include zoonotic transfer via infected animals, interpersonal spread through close contact or respiratory droplets, and vertical transmission from the Pregnant Host. (Created with the assistance of Bio-Render: https://www.biorender.com).

MPXV spreads between humans via multiple routes: direct exposure to active lesions, contact with infectious secretions (respiratory or bodily fluids), and contaminated textiles (Formenty et al., 2010; Nolen et al., 2015). Notably, intimate skin contact during sexual activity with infected genital/anal lesions serves as an efficient transmission pathway (Pan et al., 2023; Sberna et al., 2024; Svecova, 2024; Vaughan et al., 2020). The predominant mode of transmission in the 2022 outbreak has been through close contact, particularly sexual contact. Studies have shown a high prevalence of cases among men who have sex with men (MSM), suggesting that sexual transmission is a significant route (**Figure 5**) (Yan et al., 2023).

Epidemiological evidence robustly supports sexual contact as a major transmission pathway in recent outbreaks. For instance, during the 2022 global outbreak, sexual transmission was a dominant route, particularly among men who have sex with men (MSM). In one analysis, MSM accounted for 3,876 of 4,222 confirmed cases across 12 countries. This is further substantiated by the frequent detection of MPXV DNA in semen, with reported rates ranging from 13.1% to 72.4% in various cohorts (Colavita et al., 2023; Piralla et al., 2024). However, the precise mechanisms facilitating this efficient spread via mucosal contact require further definition.

The transmission of Mpox from infected gravid females to their progeny has been documented extensively within the scientific literature. Investigations have scrutinized the vertical transmission of the MPXV from maternal entities to developing fetuses, with certain studies indicating instances of fetal demise. Historical recognition of the potential for a pregnant individual infected with smallpox to convey the infection to their unborn fetus dates back to the early 1700s, with evidence suggesting that smallpox during gestation could precipitate preterm birth, miscarriage, stillbirth, or neonatal mortality (Morris et al., 2023; Schwartz et al., 2023). Mpox infection during gestation is linked to a substantial risk of detrimental outcomes. A significant proportion, 39% of cases, result in spontaneous pregnancy loss, while 23% experience intrauterine fetal mortality. Preterm parturition before 37 weeks of gestation occurs in 8% of instances. The virus demonstrates vertical transmission in 62% of cases, with 67% and 82% fetal demise rates observed in the first and second trimesters, respectively (D’Antonio et al., 2023).

Clinical investigations outcomes in four pregnant women with Clade I infection who experienced first-trimester pregnancy loss (Mbala et al., 2017). In a separate case, Ramnarayan et al. (2022) documented perinatal MPXV transmission in a UK family cluster, where an infant developed characteristic skin lesions nine days after an otherwise uncomplicated delivery. Vesicular fluid samples underwent polymerase chain reaction (PCR) analysis, which confirmed the presence of MPXV infection after a sudden decline in the health of the infant underwent 14 days of invasive ventilation and a regimen of tecovirimat and cidofovir. Following four weeks of intensive care, the infant’s condition improved significantly.

### 3.3 Reproductive health impacts of MPXV: vertical transmission, semen quality, and fertility effects

One of the most critical aspects of MPXV’s impact on reproduction is its ability to cause vertical transmission from mother to fetus, leading to adverse pregnancy outcomes. Studies in pregnant rhesus macaques, inoculated with clade IIb MPXV (the circulating strain in the Western Hemisphere), aim to understand this vertical transmission and its consequences, including fetal harm (Krabbe et al., 2025). Cases of MPXV vertical transmission have been reported in infected pregnant women, often resulting in a high viral burden in placental tissue and leading to abortion, miscarriage, stillbirth, or premature delivery (Andrieu et al., 2025). These outcomes highlight the direct pathogenic effect of the virus on the maternal-fetal interface.

Research indicates that MPXV can subvert the inflammatory response of macrophages at the maternal-fetal interface, a mechanism that likely contributes to viral persistence and adverse outcomes (Andrieu et al., 2025).

The potential for sexual transmission is underscored by the persistence of MPXV in reproductive fluids. While molecular studies have detected MPXV DNA in a high proportion (e.g., 85.7%) of semen samples from patients with acute infection (Colavita et al., 2023), it is critical to distinguish this from the presence of replication-competent virus. Definitively, viable, infectious MPXV has been successfully isolated from semen, but this appears to be confined to a narrower window than DNA detection, primarily within the first two weeks after symptom onset. Viral culture studies indicate that successful isolation from semen is most frequent within the initial 10–14 days, correlating with the period of highest clinical infectivity. The persistence of viral DNA for several weeks beyond this point, in the absence of culturable virus, suggests that the highest risk for sexual transmission likely coincides with the acute phase of illness when viable virus is present. A high viral DNA load in semen indicates significant viral replication and is a necessary precondition for the presence of infectious virus; however, its presence alone does not confirm ongoing transmissibility after the acute phase, as it may represent non-viable genetic remnants. This distinction is vital for accurate public health guidance, indicating that the duration of transmissibility via semen may be shorter than the duration of DNA positivity (Piralla et al., 2024). Indeed, a new pattern of spread among sexual networks has been described for MPXV (Castro et al., 2024), and its possible sexual transmission is a subject of ongoing investigation (Sha et al., 2024).

Beyond direct viral invasion, MPXV infection can indirectly influence reproductive health through systemic inflammation and immune responses. MPXV impairs the host’s interferon (IFN) responses, which are essential for mounting effective antiviral immunity. This impairment has broader implications, as the interferon system plays a critical role in regulating immune function and maintaining homeostasis.

The potential for MPXV to disrupt endocrine function, with major implications for long-term reproductive health, is supported by emerging direct evidence from patient biomarker studies. A recent urine metabolomics investigation of MPXV-infected patients provides direct human evidence, revealing significant disruption of steroid hormone biosynthesis pathways and lower urinary levels of key sex hormones (Savvidis et al., 2025). This hormonal dysregulation could be linked to the virus’s impact on the hypothalamic-pituitary-gonadal (HPG) axis. While direct post-mortem human data or evidence from MPXV-specific animal models on endocrine organ pathology is still limited, these human biomarker findings are consistent with patterns of endocrine disruption observed with other viral infections, such as the high prevalence of hypogonadism reported in males with severe COVID-19 (Sheikhi et al., 2025). This evidence suggests that MPXV infection can induce a state of hormone cycle imbalance, providing a plausible biological mechanism for its potential adverse effects on fertility.

Current research establishes MPXV’s capacity for vertical transmission, leading to severe adverse pregnancy outcomes such as fetal demise and abortion (Andrieu et al., 2025; Krabbe et al., 2025). The presence of viral DNA in reproductive fluids like semen warrants further investigation into its implications for sexual transmission and potential direct effects on male fertility (Colavita et al., 2023). While the direct, long-term impact on male or female fertility in survivors remains to be fully elucidated, the systemic nature of MPXV infection, its interaction with the immune system, and its potential to disrupt endocrine function suggest indirect effects on reproductive health.

### 3.4 Impact on other vulnerable populations

The impact of MPXV is disproportionately severe in vulnerable populations. Recent data from the DRC indicate that children are particularly affected, with one study reporting a 75% perinatal case fatality rate from Clade I infection (Masirika et al., 2024). The high mortality associated with Clade I mpox, particularly in vulnerable populations and regions with limited healthcare access, remains a serious issue (Srivastava et al., 2024), highlighting a stark contrast with outcomes in the 2022–2023 global Clade IIb outbreak (Mitjà et al., 2023). The enhanced human-to-human transmission of Clade Ib, including within households, facilitates this spread to children (Vakaniaki et al., 2024); Kinganda-Lusamaki et al., 2024). Furthermore, People living with HIV (PLWH), especially those with advanced immunosuppression, are at risk for severe and progressive disease (Zucker et al., 2023). Real-world evidence indicates that the collision of MPXV with HIV, particularly in individuals with untreated HIV, can lead to severe outcomes (Zucker et al., 2023), underscoring the critical need for optimized vaccination strategies, including full two-dose regimens, in this group (Back et al., 2024).

### 3.5 Implications for reproductive health

The presence of MPXV and its immune evasion mechanisms in the reproductive tract raises significant concerns. Given the sexually transmitted nature of recent MPXV outbreaks (Rothenburg et al., 2022; Sha et al., 2024), understanding how the virus interacts with the local immune environment is critical. The host’s ability to mount effective innate immune responses in the FRT and MRT is vital for preventing initial infection and subsequent dissemination. However, MPXV’s sophisticated strategies, such as inhibiting cGAS-STING signaling through OPG147 (Yi et al., 2025), or reducing IFN production via F3L (Suleman et al., 2024) and PoxS (Chan et al., 2025), underscore the challenge in controlling viral replication at these sites.

Targeted inhibition of MPXV immune modulators, like F3L, may allow for the reactivation of suppressed antiviral pathways, offering a promising direction for drug development. Furthermore, developing immunotherapies that bolster innate immune pathways in the reproductive tract could offer a proactive defense against MPXV and other sexually transmitted pathogens. This comprehensive understanding is essential for informing public health strategies, vaccine development (Bravo-Vázquez et al., 2025; Zhang et al., 2025), and the management of Mpox infections.

The detection of MPXV in ocular samples and its persistence there also underscores the virus’s ability to replicate in various tissues, potentially including those of the reproductive system (Finamor et al., 2024). Although direct evidence of MPXV causing long-term fertility impairment in males or females is still being rigorously investigated, the presence of the virus in reproductive fluids and its documented impact on fetal viability are strong indicators of a direct effect.

### 3.6 Indirect effects and broader implications for fertility

The epidemiological profile of the 2022-2024 global mpox outbreak, driven by Clade IIb MPXV, has been characterized by a pronounced concentration of cases among gay, bisexual, and other men who have sex with men (GBMSM). Surveillance data indicate a high frequency of co-infections in this population, with Human Immunodeficiency Virus (HIV) reported in over 35% of cases and other sexually transmitted infections (STIs) including *Chlamydia trachomatis*, *Neisseria gonorrhoeae*, *Treponema pallidum*, and herpes simplex virusin more than 40% of cases. Bacterial superinfection of skin lesions is also a documented complication. These concurrent infections pose a significant clinical management challenge, as they may potentiate disease severity, prolong recovery, and worsen overall outcomes. However, the specific biological and immunological interactions between MPXV and HIV, other STI pathogens, or bacteria remain poorly characterized, representing a crucial area for future research (Liu et al., 2024). The collision of MPXV with HIV, particularly in individuals with untreated HIV, can lead to severe and progressive disease (Zucker et al., 2023). These co-infections can further complicate the immune response and potentially exacerbate any adverse effects on reproductive health.

Future research should concentrate on several key areas to improve our understanding and management of Mpox: a) Identifying animal vectors, particularly nonhuman primates and African rodents, is critical to understanding the zoonotic transmission of MPXV and preventing future outbreaks. b) Investigating transmission via sexual contact and viral presence in reproductive fluids like semen is essential for refining preventive measures and public health strategies. c) Study vertical transmission during pregnancy, focusing on antiviral therapies for expectant mothers, due to potential fetal health risks. d) Further studies on MPXV’s effects on male and female fertility, including endocrine disruption and viral presence in reproductive tissues, are essential to understanding its long-term reproductive health consequences. e) Longitudinal research tracking MPXV survivors will provide insights into the long-term impact on fertility and reproductive function, guiding post-infection care and management. f) Whole-genome sequencing of MPXV across different regions will aid in monitoring viral evolution, identifying emerging variants, and informing vaccine and treatment strategies to enhance outbreak preparedness. g) Understanding how MPXV evades immune responses in the reproductive tract, through mechanisms like inhibiting cGAS-STING and IFN production, will inform the development of targeted immunotherapies. h) Research into how systemic inflammation and immune dysfunction caused by MPXV impact hormone regulation and reproductive function will be crucial for managing reproductive health risks. I) Investigating how co-infections, especially HIV, influence the severity of MPXV infection and its effects on reproductive health will help in designing integrated treatment and prevention strategies. j) Focus on whole-genome sequencing of MPXV in various outbreak regions to monitor mutations and emerging variants will facilitate the assessment of changes in transmissibility, virulence, and antiviral resistance. Comparative genomics with related poxviruses can inform vaccine and treatment strategies for enhanced outbreak preparedness.

## 4 Historical and current mpox epidemiological features

**Figure 6:**
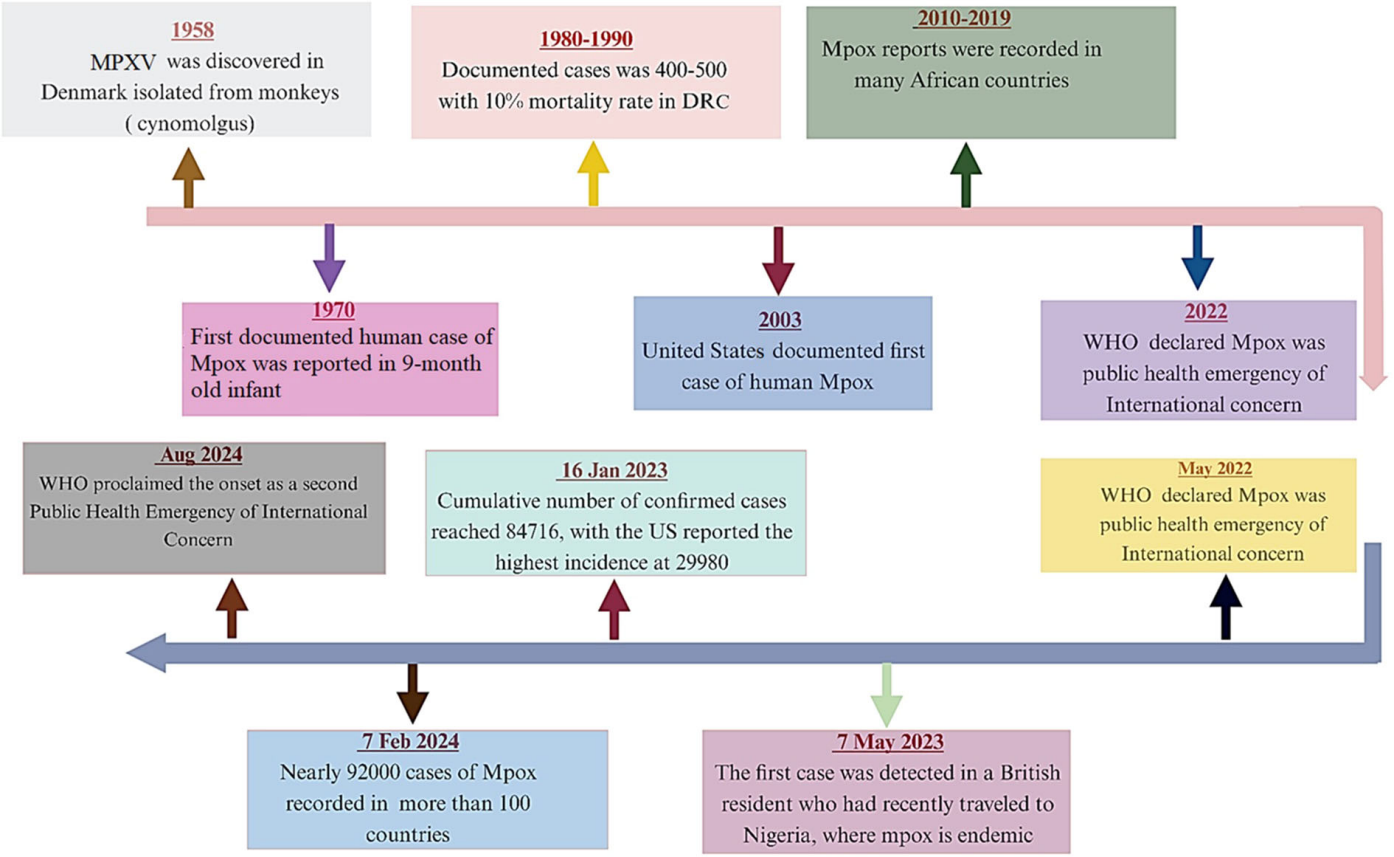
A chronological review of Mpox highlights critical discoveries and global responses over time. This figure summarizes key milestones, including the identification of the virus, the evolution of outbreaks, and the implementation of public health measures. The timeline serves to contextualize the emergence and spread of Mpox within the broader landscape of infectious disease management and emphasizes the importance of ongoing surveillance, vaccine development, and international collaboration. (Created with the assistance of Bio-Render: https://www.biorender.com).

**Figure 7:**
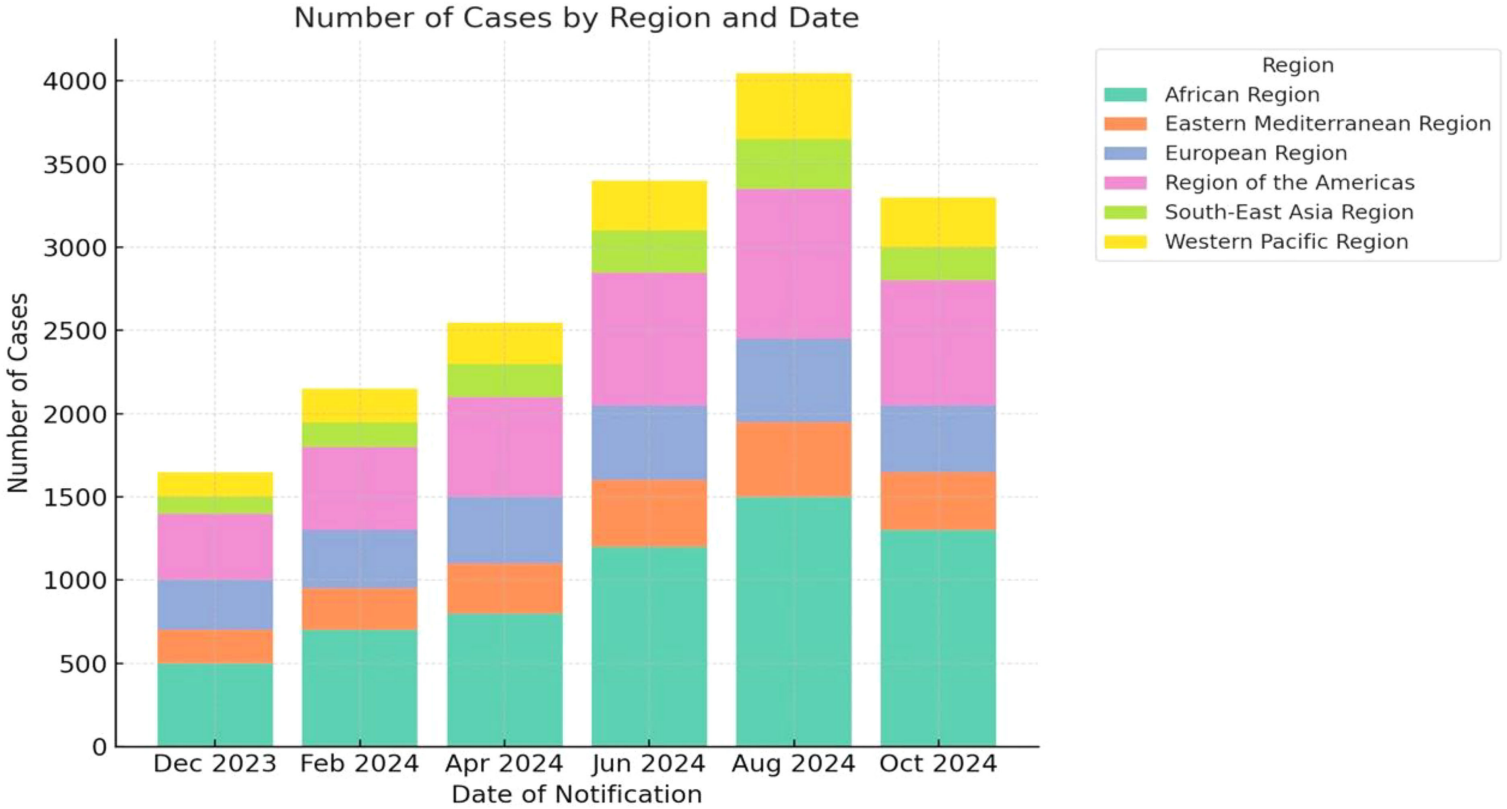
Number of Mpox cases per month reported to October 2024 (Modified Source: WHO): (https://worldhealthorg.shinyapps.io/mpx_global/#6_Genomic_epidemiology).

**Figure 8:**
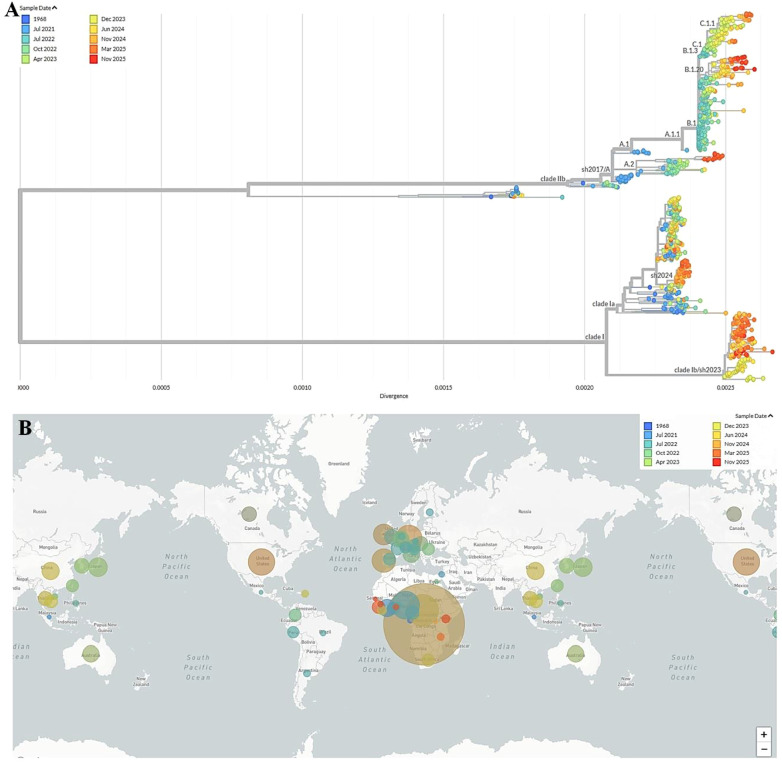
**(A)** Clade-specific genomic variations of monkeypox virus (MPXV), highlighting temporal emergence and divergence between Clade II (IIa/IIb) and Clade I (Ia/Ib). **(B)** Geographic distribution and timeline of MPXV clade-specific outbreaks, highlighting the global spread of Clade I (Ia/Ib) and Clade II (IIa/IIb) over decades. Source: (https://nextstrain.org/mpox/all-clades?c=sample_date).

Building upon its initial identification and early human cases, the epidemiological trajectory of MPXV has evolved through distinct phases, marked by intermittent outbreaks in Africa and, more recently, global spread. Following the first documented cases in the 1970s, surveillance efforts in subsequent decades revealed an increasing burden and geographic expansion within the African continent (**Figures 6**–**8**) (Marennikova et al., 1972; Fine et al., 1988; Magnus et al., 1959; Saied, 2022; Saied et al., 2022; Kumar et al., 2022).

Multiple outbreaks of human Mpox have been consistently reported in Africa, with a notable prevalence in the DRC and Nigeria (Heymann et al., 1998; Hutin et al., 2001). In the 1990s, 511 cases were documented in the DRC, with small outbreaks in equatorial West and Central Africa (Bunge et al., 2022). The initial Mpox outbreak beyond African borders occurred in 2003. This significant public health event manifested in the United States, with cases identified from May to June (Croft et al., 2007; Enserink, 2003). The outbreak originated from a zoonotic transmission chain linked to West Africa (Ghana) and involved infected prairie dogs, without reported human-to-human transmission. This marked the initial detection of human MPXV infection outside its customary African endemic (Reed et al., 2004).

The 1980s marked a notable escalation in the prevalence of Mpox cases, with documented cases surpassing 400. That decade exhibited a mortality rate of 10% and a substantial nine-fold increase in incidence. Moreover, the geographical distribution expanded to encompass four African nations, where 14 patients were identified (Bunge et al., 2022; Meyer et al., 2002). In the 1990s, 511 cases were documented in the DRC, with small outbreaks in equatorial West and Central Africa (Bunge et al., 2022). This marked the initial detection of human MPXV infection outside its customary African endemic regions (Reed et al., 2004). In 2003, the United States documented its first instance of human MPXV. While the outbreak was primarily attributed to interactions with afflicted prairie dogs, concerns persisted regarding the potential for human-to-human transmission (Fleischauer et al., 2005).

Official reports as of July 30, 2003, indicated a total of 72 human cases, comprising 37 laboratory-confirmed instances and 35 cases meeting CDC`s specified criteria (Reed et al., 2004), affected individuals had encountered prairie dogs through direct contact with an Illinois-based distributor or at locations where these animals were housed.

From 2010 to 2021, a significant number of Mpox cases were reported in various African countries, including the CFR, DRC, Cameroon, Nigeria, Liberia, and Sierra Leone. Additionally, cases were noted in the United Kingdom, Israel, and Singapore, reflecting trends observed in previous decades (Bunge et al., 2022 B; Mauldin et al., 2022). This epidemic was characterized by sustained person-to-person transmission, notwithstanding epidemiological findings suggesting sexual contact as the primary mechanism of disease propagation (Ogoina et al., 2019).

MPV case related to a traveler from Nigeria to the United States was recognized and managed bythe Texas Department of State Health Services in collaboration with the CDC (Rao AK et al., 2021). More than 200 individuals interacted with the patient, placing them at a potential risk of disease transmission. In a fortunate turn of events, there were no added instances in the early days of September, and those contacted proved to be devoid of MPV (Reed et al., 2004). However, in May 2022, the Mpox outbreak, which expanded across multiple countries on nearly every continent, was declared a public health emergency of international concern (Organization, 2024; See, 2022).

In May 2022, the UK Health Security Agency (UKHSA) documented the first instance of MPXV in the United Kingdom, marking a significant event in the context of the 2022 epidemic affecting non-endemic regions (WHO, 2022b). This initial case was identified in an individual who had recently returned to the United Kingdom from Nigeria. Subsequently, by May 12, the United Kingdom reported two additional confirmed cases and one probable recovered case. Notably, these latter cases were not linked to the initial Nigerian case and lacked any recent travel history or contact with travelers (Reed et al., 2004).

According to data from the 2022 Mpox outbreak, cases had been reported in 110 countries and territories, as documented by the WHO on January 16, 2023 (WHO, 2023). Of these, 103 locations documented their first-ever Mpox cases during this outbreak period. As of January 16, 2023, the cumulative number of confirmed cases has reached 84,716, with the United States reporting the highest incidence at 29,980 cases. Globally, 80 fatalities have been documented, with the following countries reporting the most deaths: the United States (21), Brazil (14), Peru (12), Nigeria (7), Mexico (4), Ghana (4), Spain (3), and Cameroon (3) (Reed et al., 2004). In 2022, Mpox cases were documented for the first time in non-endemic regions, including Europe and the Americas, despite the disease’s historical confinement to Central and West Africa (Khan and Perveen, 2024; Mohapatra et al., 2024).

Epidemiological investigations indicated that the majority of non-African cases were linked to recent travel from Mpox-endemic regions. These findings align with the broader trend observed in the outbreak, where most cases were epidemiologically associated with recent travel to endemic areas (CDC, 2022).

As of 7 February 2024, surveillance data indicate nearly 92,000 Mpox cases across 100+ non-endemic countries, with 156 fatalities (CDC, 2024). Concurrently, African Union reports document over 21,000 cases (including 3,000+ confirmed) in 13 member states, predominantly linked to Clades I and II, with a notable spread in Burundi, Cameroon, DRC, Ghana, Kenya, Nigeria, and South Africa (**Table 2**) (ACDC, 2024).

**Table 2:** Current MPV cases trends: aggregated data from 2022 to 2024, providing insights into the global epidemiological shifts and variations. WHO 2022–24 Mpox outbreak global trends produced on 24 December 2024 https://worldhealthorg.shinyapps.io/mpx_global/#4_Global_situation_update.

Country	Total cases	Total deaths	Reporting period	Dominant clade	Subclade distribution	CFR (%)
USA	34,349	63	May 2022 - Nov 2024	I	Ib (86%) I (14%)^b^	3.29
Brazil	13,228	16	May 2022 - Nov 2024	II	IIb (99%)	0.18
Spain	8,443	3	May 2022 - Nov 2024	II	IIb (97%)	0.12
Colombia	4,279	0	May 2022 - Nov 2024	II	IIb (95%)	0.04
France	4,371	0	May 2022 - Nov 2024	II	IIb (93%)	0.58
Germany	4,042	0	May 2022 - Nov 2024	I	Ib (100%)	0.26
Peru	3,949	23	Jun 2022 - Nov 2024	I/II	Mixed	8.35
DR Congo	10,492	45	Jan 2022 - Nov 2024	I	Ia (100%)	4.31
Argentina	1,254	2	Oct 2022 - Dec 2024	I	Ib (100%)	
Canada	1,839	0	Apr 2022 - Nov 2024	Dominant Clade	Subclade Distribution	–
Chile	1,480	3	May 2022 - Dec 2024	I	Ib (86%), I (14%)^b^	3.29
Mexico	419	35	May 2022 - Nov 2024	II	IIb (99%)	0.18
Ghana	133	4	Jan 2022 - Nov 2024	II	IIb (97%)	0.12
Uganda	784	2	Jun 2024 - Nov 2024	II	IIb (95%)	0.04
Vietnam	209	9	Oct 2022 - Nov 2024	II	IIb (93%)	0.58
Australia	1,436	0	May 2022 - Nov 2024	I	Ib (100%)	0.26
India	30	1	Jul 2022 - Nov 2024	I/II	Mixed	8.35
Italy	1,079	0	May 2022 - Nov 2024	I	Ia (100%)	4.31
Nigeria	1,006	9	Jan 2022 - Nov 2024	I	Ib (100%)	

CFR (%) represents the Case Fatality Rate, calculated as (Total Deaths/Total Cases) *x* 100%, indicating the proportion of confirmed MPXV cases that resulted in death. This metric reflects disease severity among diagnosed individuals.

The recent African CDC, August 2024, indicates alarming Mpox mortality in Africa (617 deaths among 18,737 cases; CFR 2.57%), with the DRC bearing the primary burden. This represents a dramatic rise from the <0.1% CFR reported globally during 2022-2023 (Mitjà et al., 2023). The interpretation of CFR data, particularly the stark disparities between regions, requires careful consideration of underlying surveillance systems and healthcare infrastructure. The notably high CFRs observed in some countries despite lower absolute case numbers are often not indicative of a more virulent virus but rather reflect significant surveillance bias and diagnostic limitations. In many resource-limited and rural areas, particularly within endemic African regions, surveillance systems are primarily designed to detect severe cases presenting to health facilities. This systematic bias means that mild, subclinical, or community-managed cases are frequently missed, artificially inflating the CFR by severely undercounting the true denominator of total infections (Srivastava et al., 2024). Concurrently, barriers to healthcare access, including delayed presentation, limited availability of diagnostics, and scarce critical care resources, contribute to poorer outcomes for those severe cases that are detected, further driving up the CFR. This phenomenon is starkly evident in the current Clade I outbreak in the DRC. The official figure is likely a substantial undercount, with the Africa CDC itself reporting a dramatic rise in mortality, indicating a burden that far exceeds the reported case counts (ACDC, 2024). Therefore, the high CFRs in certain settings serve as a proxy indicator for weak health systems and fragmented surveillance, rather than a pure measure of viral pathogenicity. This underscores the urgent need for investment in syndromic surveillance, decentralized testing, and equitable access to therapeutics to obtain a true picture of the disease burden and mitigate severe outcomes. As the situation intensified in gravity, the WHO Director-General declared the Mpox Outbreak a Public Health Emergency of International Concern, Geneva. 14 August 2024, proclaimed the onset as a second Public Health Emergency of International Concern (WHO, 2024). The European Centre for Disease Prevention and Control (ECDC) raised Europe’s Mpox risk level to “low” after Sweden’s first MPXV Clade Ib case on August 15, 2024, but noted minimal chances of ongoing spread (**Figure 8**) (ECDC, 2025).

Mpox cases reported monthly from December 2023 to October 2024, categorized by continent. The African Region consistently reports the highest number of cases, with a notable surge around August 2024. The European Region and the Region of the Americas show increasing cases, especially from mid-2024, with the Americas showing a noticeable rise in the latter months. The Eastern Mediterranean Region, South-East Asia Region, and Western Pacific Region also contribute to the global data, though their case numbers remain lower in comparison. This data reflects the global spread of Mpox, with significant peaks in the second half of 2024 (**Figure 6**).

## 5 Mpox clinical features

As of December 2024, epidemiological data indicated that 126 countries had reported 22,453 laboratory-confirmed cases and 67 associated fatalities (WHO, 2022–24 Mpox Outbreak Global Trends produced on 24 December 2024 https://worldhealthorg.shinyapps.io/mpx_global/#4_Global_situation_update). The transmission of MPXV occurs primarily through intimate contact with infected individuals or animals, involving exposure to skin lesions, bodily secretions, contaminated objects, and large respiratory droplets. Additionally, a previous study has confirmed the occurrence of vertical transmission (Adalja and Inglesby, 2022; Ahmed et al., 2022). While studies published between 2022 and 2023 have explored Mpox clinical symptoms within certain regions and demographic groups, none have comprehensively described the clinical manifestations in the context of the ongoing international outbreak.

Mpox is usually self-limiting, lasting 2 to 4 weeks; severe cases may require hospitalization. Clinical progression depends on prior vaccination, the patient’s immune status, and any underlying conditions. Initial symptoms, fever, myalgia, lymph node swelling, and fatigue, typically manifest within 5 days following an incubation period of up to 3 weeks (Branda et al., 2024). Mpox was predominantly found in rural children of Africa, and commonly presented with transient fever and a centrifugal, homogenous rash (Ogoina et al., 2023).But the 2022–23 Mpox outbreak revealed a demographic shift, predominantly affecting adults, especially young men. In the United States, a significant proportion of the 30,000 Mpox cases were observed in young adult men, with a notable percentage occurring among men who have MSM (Eustaquio, 2023; Singh et al., 2024).

**Figure 9:**
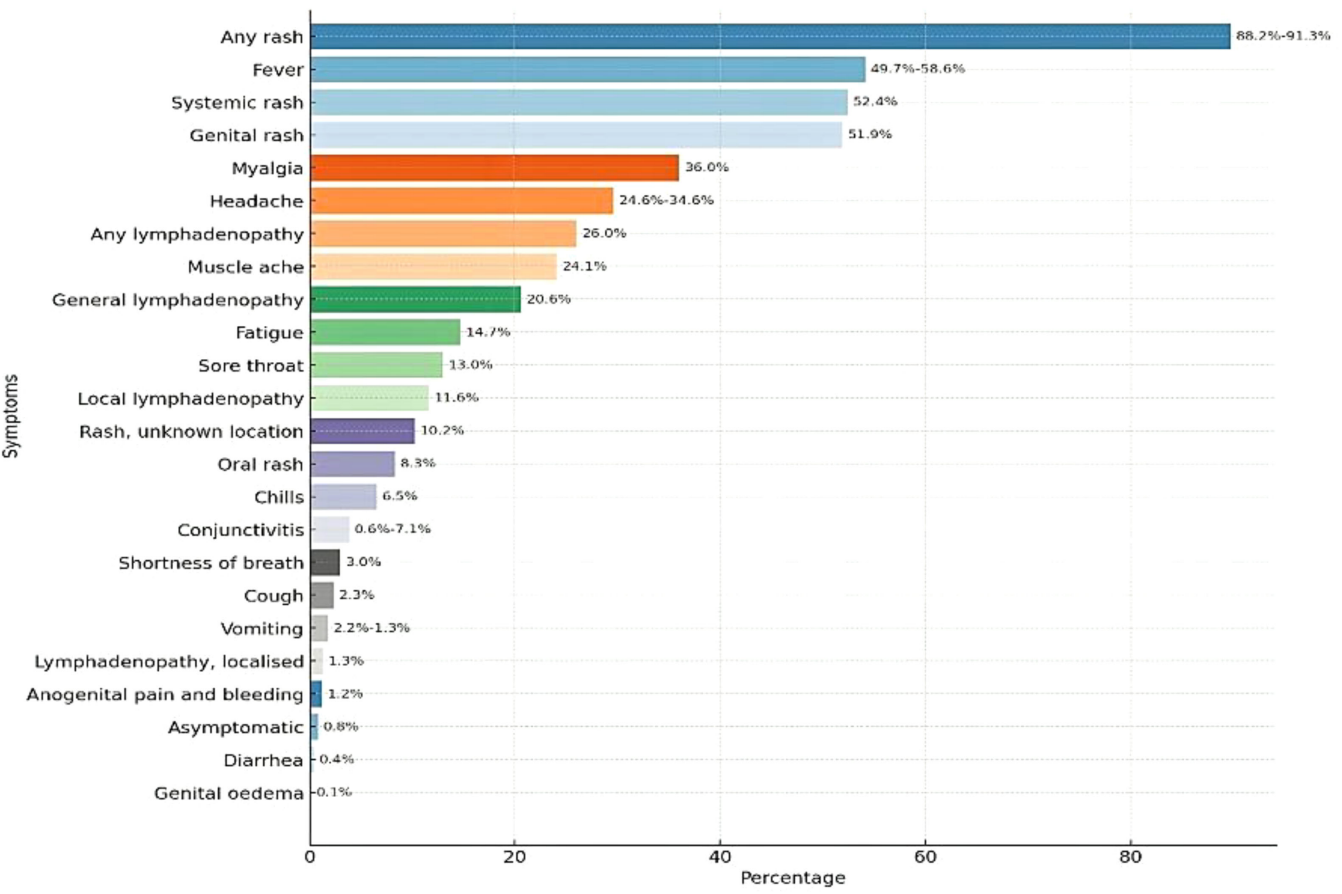
Percentage of symptoms in Mpox cases reported in 2023- Dec 2024 (https://worldhealthorg.shinyapps.io/mpx_global/).

**Figure 10:**
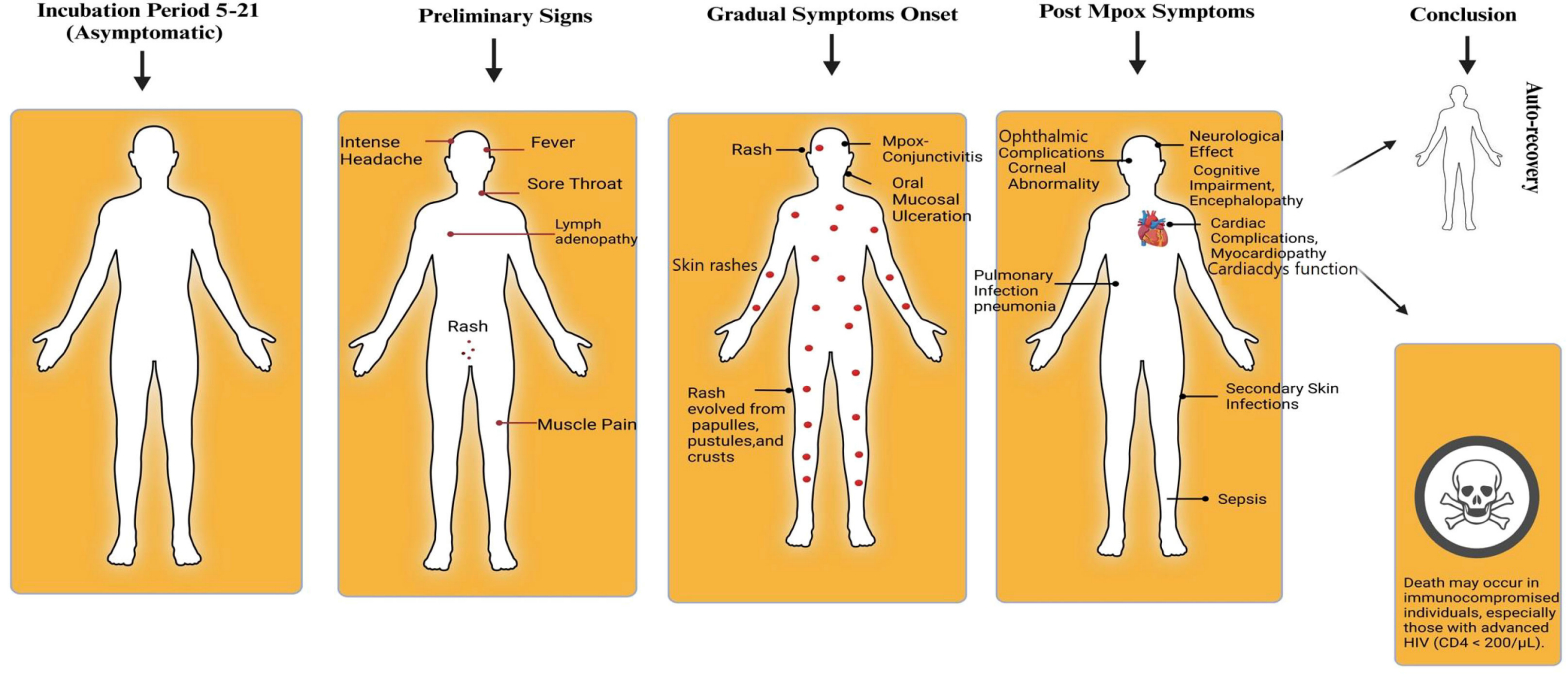
The clinical spectrum and progression of Mpox disease. The diagram outlines the four typical stages: 1) Incubation Period (5-21 days): Asymptomatic phase. 2) Preliminary Signs (Prodromal Phase): Initial onset of systemic symptoms including fever, intense headache, lymphadenopathy, sore throat, myalgia, and the initial appearance of rash. 3) Gradual Symptom Onset (Progressive Disease): The rash progresses and new complications can develop, including pustular rash (often with genital, perianal, or oral lesions), Mpox conjunctivitis, oral mucosal ulceration, pulmonary infection (pneumonia), secondary bacterial skin infections, and sepsis. 4) Post-Mpox Symptoms (Severe Complications): Potential late-stage complications occurring during or after recovery, such as neurological effects (encephalopathy, cognitive impairment) and cardiac complications (myocardiopathy, cardiac dysfunction). A fatal risk is associated with immunocompromised individuals, particularly those with advanced HIV (CD4 count < 200/µL).

The latest outbreak featured lesions mostly on the face, genitalia, and mucosal surfaces, unlike the prior widespread rash distribution. Certain cases presented lesions localized to specific regions, such as the genital area, in contrast to the broader distribution in earlier outbreaks (Abou Chakra et al., 2023; Pérez-Martín et al., 2022). Mpox typically begins with fever (50%-58%), Skin rash (88.2- 91.3%), headache (24.6%-34.6%), muscle aches (24.1%), fatigue (14.7%), swollen lymph nodes (26.0%), chills (6.5%), myalgia (36.0%), nausea or vomiting (2.5-13%), conjunctivitis (0.6%-7.1%), cough (2.3%), shortness of breath (3.3%), sore throat (13.1%), diarrhea (0.4%), and genital discomfort (1.2%) (Yon et al., 2023; Lim et al., 2024). The rash evolves from papules to vesicles, pustules, and crusts, often causing pain (**Figures 9** and **10**) (Kasecker et al., 2024; Pourriyahi et al., 2023).

## 6 Mpox diagnostic protocols

Clinical symptoms alone are insufficient for accurately diagnosing Mpox, making molecular assays and patient specimen testing essential for confirmation. These diagnostic tests play a critical role in identifying *Orthopoxvirus* infections with precision. MPXV and other O*rthopoxviruses* can be accurately determined through advanced diagnostic methods using patient-derived clinical specimens (Kulesh et al., 2004). Mpox should be included in the differential diagnoses of patients presenting with a suspicious rash, particularly when accompanied by systemic symptoms (Minhaj et al., 2022).

Identifying Mpox cases requires careful evaluation of travel history to affected areas and direct or indirect exposure to infected individuals (Minhaj et al., 2022). A comprehensive approach to MPXV infections can be divided into clinically approved diagnostic assays and research protocols.

### 6.1 Clinically approved diagnostic assays

The most precise method for MPXV diagnosis involves PCR examination of lesion-derived specimens (WHO, 2022a; Altindis et al., 2022). Clinical laboratories typically utilize assays that have received regulatory approval, such as those authorized by the US FDA. Other widely accepted techniques include serological evaluation of IgG and IgM via ELISA.

### 6.2 Research protocols and assays

In contrast, research laboratories may employ techniques such as viral culture, electron microscopy, immunohistochemistry, and CRISPR-Cas12 for diagnosing MPXV. However, these approaches require access to advanced laboratory infrastructure and adherence to stringent biosafety regulations (Harapan et al., 2022; McCollum and Damon, 2014) It is essential to note that clinical labs do not perform extensive viral cultures and electron microscopy for routine diagnosis.

Cell culture-based virus detection, the isolation of MPXV from clinical specimens, particularly skin lesions, is the gold standard for confirming active infection by demonstrating viral replication. Viral propagation in cell cultures enables direct observation of replication dynamics, ensuring high specificity. However, this method is labor-intensive, requires specialized biosafety facilities, and is impractical for rapid outbreak response (Piralla et al., 2024; Resman Rus et al., 2024).

Electron microscopy (EM) provides rapid MPXV identification and distinguishes it from other viruses. Though useful alongside nucleic acid amplification techniques (NAT), its use has declined due to more sensitive molecular assays. Its need for specialized labs and expertise limits accessibility (Gelderblom and Madeley, 2018).

Immunohistochemistry (IHC) detects MPXV-specific proteins in skin lesions using monoclonal antibodies against viral antigens like A29 and A35, confirming infection and differentiating Mpox from clinically similar diseases, such as cutaneous syphilis, by detecting specific viral proteins in tissue samples (Ng et al., 2025; Shi et al., 2025).

CRISPR-Cas12 system enables MPXV detection by fluorescence, even when viral titers are low (Liang et al., 2024; Sui et al., 2022). Its reliability and convenience make it an effective tool for rapid diagnosis, including asymptomatic cases, aiding in timely intervention to curb viral transmission (Zhou and Chen, 2023).

ELISA and immunofluorescence facilitate high-throughput IgG and IgM detection for epidemiological studies and can be adapted for BSL-2 using attenuated *Orthopoxviruses* like vaccinia virus (Grossegesse et al., 2023). A peptide-based ELISA demonstrated ~86% sensitivity and ~90% specificity, aiding in distinguishing prior vaccination from recent MPXV infection (Pang et al., 2024; Taha et al., 2023), but high antigenic overlap among orthopoxviruses limits the accuracy of serological tests in differentiating MPXV from smallpox vaccination, requiring improved diagnostic methods (Hunt et al., 2024; Lee et al., 2023).

### 6.3 Treatment

MPXV infection is typically self-limiting, with most individuals recovering without medical intervention. However, several antiviral agents, originally approved for smallpox treatment in animal models, have shown potential efficacy against Mpox and may be considered in severe cases (Adler et al., 2022). Supportive care remains the primary approach for Mpox management, addressing symptoms, hydration, and bacterial superinfections, as no specific antiviral treatment is endorsed (Huang et al., 2022; “Infezioni in Medicina,” 2023).

### 7.1 Tecovirimat

#### 7.1.1 Drug chemistry

Tecovirimat is an antiviral agent used primarily against Orthopoxviruses, including MPXV. It is known for its structural target specificity, focusing on the VP37 protein encoded by the F13 gene.

#### 7.1.2 Drug mechanism of action

Tecovirimat inhibits viral replication by promoting the dimerization of the F13 phospholipase, which is crucial for viral egress. The structural conservation of the VP37 protein across Orthopoxviruses enhances its effectiveness against various MPXV strains (Prasetyo et al., 2024).

#### 7.1 Efficacy

The efficacy of Tecovirimat has been recently reassessed. While in vitro studies show potent inhibition, a pivotal randomized controlled trial (PALM007) in the DRC found no significant clinical benefit of Tecovirimat over placebo for the primary endpoint of time to lesion resolution in Clade I infection, highlighting a critical efficacy gap. Concurrently, emerging resistance, particularly in immunocompromised hosts, underscores the need for novel strategies. Promising in vitro data suggest combination therapies with host-targeting agents like Mycophenolate mofetil (MMF) can produce strong synergistic effects and raise the genetic barrier to resistance (Witwit et al., 2024, 2025).

This emerging resistance is primarily conferred by specific mutations within the viral F13L gene, which encodes the VP37 protein targeted by Tecovirimat. Key mutations identified in clinical and surveillance studies include N267del (a deletion at asparagine 267) and A184T (a substitution of alanine for threonine), which are located at the drug-binding dimer interface and prevent the stable protein-protein interactions necessary for the drug’s activity. The current global prevalence of these resistant strains is officially documented as low (constituting less than 1% of sequenced cases in the United States as of early 2024). However, the confirmed person-to-person transmission of a Tecovirimat-resistant MPXV strain harboring both the N267del and A184T mutations among patients without prior drug exposure marks a significant escalation of the threat (Witwit et al., 2024, 2025). This development underscores the non-negotiable need for systematic F13L gene sequencing in surveillance programs and accelerates the urgency for developing and approving next-generation antivirals with distinct mechanisms of action.

#### 7.1.4 Safety profile

The safety profile of Tecovirimat is generally favorable, with common side effects being mild, such as fatigue and nausea. Rare adverse effects include liver enzyme abnormalities and potential psychiatric issues (Shabil et al., 2024).

### 7.2 Cidofovir

#### 7.2.1 Drug chemistry

Cidofovir is a nucleotide analog ((S)−1-(3-hydroxy-2-phosphono-methoxypropyl)), targeting the DNA polymerase complex of viruses, particularly the MPXV.

#### 7.2.2 Drug mechanism of action

Cidofovir disrupts viral replication by inhibiting dCTP incorporation into viral DNA, effectively halting the replication process (Sui et al., 2022).

#### 7.2.3 Efficacy

While cidofovir shows substantial antiviral potential against Mpox, its clinical use is tempered by nephrotoxicity.

#### 7.2.4 Safety profile

The use of cidofovir is complicated by its nephrotoxic effects, necessitating concurrent hydration and the administration of probenecid to mitigate renal damage (Braddick and Singh, 2024; Duong et al., 2024).

### 7.3 Brincidofovir

#### 7.3.1 Drug chemistry

Brincidofovir is a lipid-modified derivative of cidofovir designed to enhance oral absorption and diminish renal toxicity.

#### 7.3.2 Drug mechanism of action

It similarly targets viral DNA polymerase to inhibit poxvirus replication, effectively impeding viral proliferation.

#### 7.3.3 Efficacy

In animal studies, brincidofovir demonstrated a significant reduction in MPXV clade II replication, particularly in the respiratory tract (Prévost et al., 2024).

#### 7.3.4 Safety profile

Clinical findings have indicated a generally safe profile for brincidofovir, with mild gastrointestinal effects and temporary alterations in liver function noted (Huston et al., 2023).

## 8 Vaccination and prevention

Per CDC guidelines, the absence of an MPXV-specific vaccine necessitates reliance on smallpox immunization. To control transmission, vaccinating high-risk populations, including MSM, is crucial (MacIntyre and Grulich, 2022) Recent outbreaks highlight the need for equitable vaccine access and public health initiatives focused on awareness, destigmatization, and timely care (O’Neil et al., 2024).

MPXV vaccination is endorsed for high-risk populations; studies show this could effectively eliminate the virus in semi-endemic areas, but efficacy in fully endemic areas remains limited (Bankuru et al., 2020; Khan et al., 2024; Back et al., 2024). Currently, two vaccines are FDA-approved for Mpox prevention: A) JYNNEOS (live, replication-deficient vaccinia virus, Bavarian Nordic; also known as IMVAMUNE and IMVANEX) and B) ACAM2000 (live replicating vaccinia virus, Emergent BioSolutions). Vaccines in development include mRNA-1769 (mRNA platform), LC16m8 (attenuated replicating strain), and BNT166a (mRNA platform). When vaccination efforts are stopped or withheld, it is imperative to understand the associated risks to high-risk populations, and alternative measures must be implemented to control transmission (https://clinicaltrials.gov/study/NCT05745987, JYNNEOS & https://clinicaltrials.gov/study/NCT02977715). While approved vaccines are already deployed for pre/post-exposure prophylaxis, developmental candidates aim to enhance immunity breadth and accessibility. All show potential to prevent infection or reduce severity. Monoclonal antibodies and antivirals are also being researched as targeted treatment options (Khan et al., 2024), with the identification of targeted antivirals and improvement of access to vaccines and antivirals, especially in underserved areas, as a priority (O’Neil et al., 2024).

The MVA-BN vaccine is a cornerstone of prevention. A large German cohort study confirmed its safety and estimated the effectiveness of a single dose at 57.8% overall, with significantly reduced effectiveness in people living with HIV, reinforcing the need for potentially boosted schedules in immunocompromised populations (Back et al., 2024). WHO recommends the MVA-BN for individuals at high risk for MPXV exposure, including healthcare workers and laboratory workers working with *Orthopoxviruses* (Vaccin Contre La Variole et La Mpox MVA-BN (Modified Vaccinia Ankara - Bavarian Nordic), 2025).

The LC16m8 vaccine was developed in Japan as a safer alternative to prevent and control smallpox outbreaks; it is based on a live attenuated vaccinia virus (VACV) and has been licensed in Japan since 1975 to provide immunity against smallpox (Stovba et al., 2024). The LC16m8 vaccine is derived from the Lister strain and is characterized by a mutation in the B5R gene to enhance safety. The LC16m8 vaccine is considered a third-generation vaccine and, in 2024, was evaluated and provided evidence of effectiveness and safety in preventing Mpox in high-risk populations (Okumura et al., 2024). While the potential for Mpox vaccination exists, challenges around access, distribution, and hesitancy remain within the context of public health efforts to improve global vaccination opportunities and equity (Zinnah et al., 2024).

## 9 Public health strategies: successes and hurdles

Public health authorities are focusing on targeted and available vaccinations. They prioritize educating and engaging the community about stigma and misinformation regarding vaccines. By informing the community about vaccine availability and safety, these efforts aim to increase awareness and promote informed decision-making about vaccination and eventual (Biesty et al., 2024).

Challenges related to vaccine hesitancy, logistical issues, and production capacity continue to impact Mpox response efforts in vulnerable areas. Ongoing research into Mpox epidemiology and vaccine effectiveness will inform and adapt future immunization strategies as the virus evolves.

The continued development of targeted vaccines, along with surveillance, education, and international coordination, is critical for the prevention and control of Mpox on a global scale (Koppe et al., 2024; Ouyang et al., 2024).

Surveillance systems, especially systems with real-time disease tracking using data collection and reporting systems, are vital for the early detection and management of infectious disease outbreaks. Surveillance systems depend on strong data collection and reporting systems that can take advantage of cutting-edge technologies, such as big data, IoT, AI, and GIS, which can use collected raw data to create spatial visualizations that map the spread of an infection, allowing for the detection of patterns that will inform timely and efficient interventions in the management of an outbreak (Parker and Buller, 2013; Anglemyer et al., 2020; B R, 2025; Chaturvedi et al., 2024). The effectiveness of contact tracing can be improved by extending its reach to include indirect exposure to persons diagnosed with Mpox (Bansal et al., 2025). Given the importance of accuracy and expedience, particularly in the absence of available pre-exposure vaccination, a delay in follow-up can critically impact the transmission of Mpox (Chaturvedi et al., 2024). Literature respecting other public health emergencies has shown that successful management of an outbreak is contingent on a well-structured surveillance system that adequately provides for efficient data sharing and contact tracing efforts (Gashema et al., 2024). A surveillance system would necessitate multisectoral collaboration at the local, national, and global level, as such surveillance would benefit from a stronger healthcare infrastructure to support data collection efforts, protocols for diagnostic testing, and a workforce that warrants rigorous training to combat a future outbreak. Despite advancements in technology and contact tracing systems, there continue to be challenges in efforts to communicate the data in a timely way, such as inconsistent reporting, lack of privacy concerns by some individuals or organizations, and limited real-time connectivity to community members located in remote areas. These barriers must be overcome through collaborative planning and improvements to the systems involved in communicating data to enhance the capacity to respond to outbreaks. Furthermore, gaining public trust and fighting misinformation are also critical to mitigating Mpox infections (B R, 2025; Gashema et al., 2024).

## 10 Future perspectives

Future research about Mpox should prioritize the following factors: enhanced surveillance, improved diagnostics, and forward-thinking antiviral therapies. The ability to produce vaccines that can be rapidly and effectively deployed in outbreak response is critical (Li et al., 2024). Outbreak readiness is also predicated on proactive preparedness, learning from specific scenarios through simulation-based training, and stockpiling medical supplies and resources. When combined with an emphasis on system-wide health system strengthening, this allows for preparedness and prompt crisis response. Previous lessons learned during the COVID-19 pandemic show that responding to more frequent Mpox outbreaks could be a growing challenge. We should prioritize preventive strategies, especially for vulnerable communities, aiming to lessen the impact on public health systems (Yang, 2022). Mpox had previously only been reported in Africa. The rising prevalence of the Mpox virus has already added burdens to healthcare system budgets and contributed to a social stigma that will require effective prevention and response. Vaccination access must increase in areas of high burden and among populations deemed at risk, including those living with HIV, to effectively limit transmissibility (Alakunle and Okeke, 2022; Biesty et al., 2024; Ouyang et al., 2024; Koppe et al., 2024; Anglemyer et al., 2020; B R, 2025; Bansal et al., 2025; Gashema et al., 2024; Yang, 2022; Nakoune and Olliaro, 2022; Karbalaei and Keikha, 2022). Vaccine access and placement must also consider risks among those who are pregnant and for individuals who are HIV positive, thinking ethically about access to vaccination (Jamieson et al., 2005; Hooper, C., 1998). While vaccines for Mpox are still emerging, researchers have evaluated several candidate vaccines that target relevant viral proteins (A27L, A33R, L1R, B5R). These vaccine candidates have provided initial evidence of immune responses among animals (Wang et al., 2024; Zeng et al., 2023). Continued research and investment are fundamental to the development and mass distribution of vaccine candidates and further to global readiness for future outbreaks.

## 11 Limitations of the current evidence base

While this review synthesizes the most current understanding of the evolving Mpox threat, it is important to acknowledge the limitations inherent in the available body of literature, which inevitably shape our conclusions. A primary constraint is the heavy reliance on observational studies, case series, and cross-sectional analyses generated during the rapid outbreak response from 2022 onward. While these studies are invaluable for timely insights, they often lack the controlled design of prospective cohort studies or randomized trials, potentially introducing biases in estimates of risk factors, clinical spectrum, and therapeutic efficacy. Furthermore, the global data are marked by heterogeneity; reporting standards, diagnostic protocols, and public health surveillance capacities vary significantly between non-endemic and endemic countries, particularly within Africa. This can lead to underreporting, incomplete clinical characterization, and a fragmented understanding of the true burden and presentation of the disease in different geographical and socioeconomic contexts.

Another significant limitation is the demographic focus of a large portion of the published reports from the 2022–2024 outbreak, which predominantly involved men who have sex with men (MSM) in high-income nations. This focus, while crucial for understanding the outbreak’s dynamics in those networks, may limit the generalizability of findings related to transmission routes, clinical features, and immune responses to other populations, including women, children, and non-sexual transmission networks in endemic regions. Finally, the long-term implications of MPXV infection, especially concerning reproductive health, fertility, and the duration of immune protection, remain poorly characterized due to the relatively short time since the major global outbreak began. The evidence on these critical issues is currently based on a limited number of case reports and short-term follow-up studies.

To manage these limitations and build a more robust evidence base, future research must prioritize well-designed longitudinal cohort studies that track diverse patient populations over time. There is a pressing need for standardized, global case reporting forms to ensure data comparability. Prospective studies are urgently required to definitively assess the impact on male and female fertility, the kinetics of viral shedding in various bodily fluids, and the durability of vaccine-induced immunity. Finally, strengthening diagnostic and surveillance infrastructure in endemic countries is paramount to ensure a more equitable and complete picture of the virus’s evolution and ecology, moving beyond a crisis-driven narrative to a sustainable, global research effort.

## 12 Conclusions

The present work outbreak signifies a paradigm shift in the global threat of this pathogen, moving from a geographically limited zoonosis to a virus capable of sustained global human-to-human transmission. Our comprehensive review concludes that the emergence of novel lineages, particularly the rapidly spreading Clade Ib in the DRC characterized by APOBEC3-mediated mutations and enhanced human-to-human transmissibility, underscores a dynamic and concerning evolutionary landscape. This outbreak has revealed the virus’s sophisticated immune evasion tactics, specifically its interference with type I interferon signaling and modulation of pro-inflammatory cytokines, which complicates infection control. Critically, the transmission paradigm has expanded to prominently include sexual transmission, leading to significant and previously underrepresented clinical consequences, including vertical transmission with high rates of fetal loss, viral persistence in semen, and potential long-term impacts on reproductive health, often exacerbated in a syndemic with HIV. While countermeasures exist with PCR as a diagnostic cornerstone, antivirals like tecovirimat demonstrating efficacy, and vaccines like MVA-BN providing protection their global deployment remains inequitable. Therefore, we urgently recommend the establishment of coordinated international genomic surveillance networks to monitor variant emergence in real-time, a prioritized research agenda focused on fertility impacts and novel antiviral combinations to preempt resistance, the strengthening of public health systems through the integration of Mpox response into sexual health programs and the combating of stigma, and a concerted acceleration in developing next-generation pan-orthopoxvirus vaccines and broad-spectrum antivirals. Implementing these proactive, scientifically rigorous, and equitable strategies is imperative to mitigate the current crisis and build a durable defense against the ongoing threat of MPXV and future emergent orhopoxviruses.

## References

[B1] Abou Chakra M. Duquesne I. Bou Yassine A. Hanna E. Barry Delongchamps N. Jida M. . (2023). Isolated monkeypox genital lesions. Scand. J. Urol. 57, 115–116. doi: 10.1080/21681805.2022.2138534, PMID: 36324192

[B2] Adalja A. Inglesby T. (2022). “ A novel international monkeypox outbreak,” in Ann. Intern. Med. 175, 1175–1176. doi: 10.7326/M22-1581, PMID: 35605243

[B3] Adler H. Gould S. Hine P. Snell L. B. Wong W. Houlihan C. F. . (2022). Clinical features and management of human monkeypox: a retrospective observational study in the UK. Lancet Infect. Dis. 22, 1153–1162. doi: 10.1016/S1473-3099(22)00228-6, PMID: 35623380 PMC9300470

[B4] Africa Centres for Disease Control and Prevention (2024). Africa CDC Epidemic Intelligence Weekly Report. Available online at: https://africacdc.org/download/africa-cdc-weekly-event-based-surveillance-report-august-2024/ (Accessed September 7, 2024).

[B5] Ahmed S. K. Rashad E. A. A. Mohamed M. G. Ravi R. K. Essa R. A. Abdulqadir S. O. . (2022). The global human monkeypox outbreak in 2022: An overview. Int. J. Surg. 104, 106794. doi: 10.1016/j.ijsu.2022.106794, PMID: 35918003 PMC9624120

[B6] Alakunle E. Kolawole D. Diaz-Cánova D. Alele F. Adegboye O. Moens U. . (2024). A comprehensive review of monkeypox virus and mpox characteristics. Front. Cell. Infect. Microbiol. 14. doi: 10.3389/fcimb.2024.1360586, PMID: 38510963 PMC10952103

[B7] Alakunle E. F. Okeke M. I. (2022). Monkeypox virus: a neglected zoonotic pathogen spreads globally. Nat. Rev. Microbiol. 20, 507–508. doi: 10.1038/s41579-022-00776-z, PMID: 35859005 PMC9297671

[B8] Albin J. S. Lazarus J. E. Hysell K. M. Rubins D. M. Germaine L. Dugdale C. M. . (2022). Development and implementation of a clinical decision support system tool for the evaluation of suspected monkeypox infection. J. Am. Med. Inf. Assoc. 29, 2124–2127. doi: 10.1093/jamia/ocac151, PMID: 36036367 PMC9667162

[B9] Altindis M. Puca E. Shapo L. (2022). Diagnosis of monkeypox virus – an overview. Travel Med. Infect. Dis. 50, 102459. doi: 10.1016/j.tmaid.2022.102459, PMID: 36109000 PMC9534096

[B10] Altmann D. M. (2023). The COVID-19 immunology masterclass enters its fourth year. Nat. Immunol. 24, 201–202. doi: 10.1038/s41590-022-01393-x, PMID: 36604546

[B11] Andrieu J. Valade M. Wurtz N. Lebideau M. Bretelle F. La Scola B. . (2025). Monkeypox virus subverts the inflammatory response of macrophages at the maternal-fetal interface. J. Med. Virol. 97, e70412. doi: 10.1002/jmv.70412, PMID: 40400454 PMC12096145

[B12] Anglemyer A. Moore T. H. Parker L. Chambers T. Grady A. Chiu K. . (2020). Digital contact tracing technologies in epidemics: a rapid review. Cochrane Database Syst. Rev. CD013699. doi: 10.1002/14651858.CD013699, PMID: 33502000 PMC8241885

[B13] Arndt W. D. Cotsmire S. Trainor K. Harrington H. Hauns K. Kibler K. V. . (2015). Evasion of the innate immune type I interferon system by monkeypox virus. J. Virol. 89, 10489–10499. doi: 10.1128/JVI.00304-15, PMID: 26246580 PMC4580173

[B14] (2025). Vaccin contre la variole et la mpox MVA-BN (Modified Vaccinia Ankara - Bavarian Nordic) ( World Health Organization). doi: 10.2471/B09279

[B15] Back S. Knox B. Coakley C. Deltour N. Jacquot E. Raad H. . (2024). Effectiveness and safety of the MVA–BN vaccine against mpox in at-risk individuals in the United States (USMVAc). Vaccines 12, 651. doi: 10.3390/vaccines12060651, PMID: 38932380 PMC11209565

[B16] Bankuru S. V. Kossol S. Hou W. Mahmoudi P. Rychtář J. Taylor D. (2020). A game-theoretic model of monkeypox to assess vaccination strategies. PeerJ 8, e9272. doi: 10.7717/peerj.9272, PMID: 32607280 PMC7316080

[B17] Bansal J. Kumar A. Kumar A. Khan A. Abdeljawad T. (2025). Investigation of monkeypox disease transmission with vaccination effects using fractional order mathematical model under Atangana-Baleanu Caputo derivative. Model. Earth Syst. Environ. 11, 40. doi: 10.1007/s40808-024-02202-0

[B18] Beer E. M. Rao V. B. (2019). A systematic review of the epidemiology of human monkeypox outbreaks and implications for outbreak strategy. PloS Negl. Trop. Dis. 13, e0007791. doi: 10.1371/journal.pntd.0007791, PMID: 31618206 PMC6816577

[B19] Berhanu A. Wilson R. L. Kirkwood-Watts D. L. King D. S. Warren T. K. Lund S. A. . (2008). Vaccination of BALB/c mice with *Escherichia coli* -expressed vaccinia virus proteins A27L, B5R, and D8L protects mice from lethal vaccinia virus challenge. J. Virol. 82, 3517–3529. doi: 10.1128/JVI.01854-07, PMID: 18199639 PMC2268497

[B20] Biesty C. P. Hemingway C. Woolgar J. Taylor K. Lawton M. D. Waheed M. W. . (2024). Community led health promotion to counter stigma and increase trust amongst priority populations: lessons from the 2022–2023 UK mpox outbreak. BMC Public Health 24, 1638. doi: 10.1186/s12889-024-19176-4, PMID: 38898512 PMC11188168

[B21] Bordi L. D’Auria A. Frasca F. Mazzotta V. Mazzetti P. Fracella M. . (2024). MPXV infection impairs IFN response but is partially sensitive to IFN-γ antiviral effect. Med. Microbiol. Immunol. 213, 25. doi: 10.1007/s00430-024-00808-w, PMID: 39527317

[B22] B R Y. (2025). Real time mapping of epidemic spread. Interantional J. Of Sci. Res. In Eng. And Manage. 09, 1–9. doi: 10.55041/IJSREM40811

[B23] Braddick M. Singh K. P. (2024). Therapeutic agents for the treatment of human mpox. Curr. Opin. Infect. Dis. 37, 518–525. doi: 10.1097/QCO.0000000000001069, PMID: 39382085

[B24] Branda F. Romano C. Ciccozzi M. Giovanetti M. Scarpa F. Ciccozzi A. . (2024). Mpox: an overview of pathogenesis, diagnosis, and public health implications. J. Clin. Med. 13, 2234. doi: 10.3390/jcm13082234, PMID: 38673507 PMC11050819

[B25] Bravo-Vázquez L. A. Bernal-Vázquez D. Duttaroy A. K. Paul S. (2025). Current status of next-generation vaccines against mpox virus: a scoping review. Front. Pharmacol. 16. doi: 10.3389/fphar.2025.1533533, PMID: 40356988 PMC12066571

[B26] Breman J. G. Kalisa-Ruti Steniowski M. V. Zanotto E. Gromyko A. I. Arita I. (1980). Human monkeypox 1970-79. Bull. World Health Organ. 58, 165–182., PMID: 6249508 PMC2395797

[B27] Buerke M. Böttger P. Lemm H. (2025). Mpox – diagnostik, therapie, immunisierung und prognose. Medizinische Klinik - Intensivmedizin Und Notfallmedizin 120, 141–144. doi: 10.1007/s00063-024-01198-w, PMID: 39503781

[B28] Buller R. M. Palumbo G. J. (1991). Poxvirus pathogenesis. Microbiol. Rev. 55, 80–122. doi: 10.1128/mr.55.1.80-122.1991, PMID: 1851533 PMC372802

[B29] Bunge E. M. Hoet B. Chen L. Lienert F. Weidenthaler H. Baer L. R. . (2022). The changing epidemiology of human monkeypox—A potential threat? A systematic review. PloS Negl. Trop. Dis. 16, e0010141. doi: 10.1371/journal.pntd.0010141, PMID: 35148313 PMC8870502

[B30] Butler K. Banday A. R. (2023). APOBEC3-mediated mutagenesis in cancer: causes, clinical significance and therapeutic potential. J. Hematol. Oncol. 16, 31. doi: 10.1186/s13045-023-01425-5, PMID: 36978147 PMC10044795

[B31] Castro G. M. Sicilia P. E. Willington A. López L. Poklepovich T. Campos J. . (2024). Emergence of monkeypox virus in central Argentina: Epidemiological features and first complete genome sequences in the country. Rev. Argent. Microbiol. 56, 276–280. doi: 10.1016/j.ram.2024.05.003, PMID: 39034190

[B32] Centers for Disease Control and Prevention (CDC) Monkeypox. Available online at: https://www.cdc.gov/poxvirus/monkeypox/index.html.

[B33] Centers for Disease Control and Prevention (CDC) Monkeypox: isolation and infection control: home. Available online at: https://stacks.cdc.gov/view/cdc/119776 (Accessed April 19, 2024).

[B34] Centers for Disease Control and Prevention (CDC) (2003a). Monkeypox: report of cases in the United States. Available online at: http://www.cdc.gov/od/oc/media/mpv/cases.htm (Accessed January 10, 2025).

[B35] Centers for Disease Control and Prevention (CDC) (2003b). Update: multistate outbreak of monkeypox—Illinois, Indiana, Kansas, Missouri, Ohio, and Wisconsin 2003. MMWR Morb. Mortal. Wkly. Rep. 52, 642–646. Available online at: https://www.cdc.gov/mmwr/preview/mmwrhtml/mm5227a5.htm. 12855947

[B36] Centers for Disease Control and Prevention (CDC) (2022). monkeypox outbreak global map. Available online at: https://www.cdc.gov/poxvirus/monkeypox/response/2022/world-map.html2022 (Accessed January 15, 2025).

[B37] Centers for Disease Control and Prevention (CDC) (2022–2023a). Mpox Outbreak Global Map. Data as of 7 February 2024. Available online at: https://archive.cdc.gov/\/details?url=https://www.cdc.gov/poxvirus/mpox/response/2022/world-map.html (Accessed March 5, 2024).

[B38] Centers for Disease Control and Prevention (CDC) (2023). Case Definitions for Use in the 2022 Mpox Response. Available online at: https://www.cdc.gov/mpox/hcp/casedefinitions/?CDC_AAref_Val=https://www.cdc.gov/poxvirus/mpox/clinicians/case-definition.html (Accessed March 6, 2024).

[B39] Centers for Disease Control and Prevention . (2024). Monkeypox in the United States and Around the World: Current Situation. U.S. Department of Health and Human Services. Available online at: https://www.cdc.gov/monkeypox/situation-summary/index.html.

[B40] Chan P. Ye Z.-W. Zhao W. Ong C.-P. Sun X.-Y. Cheung P.-H. H. . (2025). Mpox virus poxin-schlafen fusion protein suppresses innate antiviral response by sequestering STAT2. Emerg. Microbes Infect. 14, 2477639. doi: 10.1080/22221751.2025.2477639, PMID: 40066622 PMC11921170

[B41] Chaturvedi M. Rodiah I. Kretzschmar M. Scholz S. Lange B. Karch A. . (2024). Estimating the relative importance of epidemiological and behavioural parameters for epidemic mpox transmission: a modelling study. BMC Med. 22, 297. doi: 10.1186/s12916-024-03515-8, PMID: 39020322 PMC11256368

[B42] Chen Z. Eggerman T. L. Bocharov A. V. Baranova I. N. Vishnyakova T. G. Patterson A. P. (2021). APOBEC3-induced mutation of the hepatitis virus B DNA genome occurs during its viral RNA reverse transcription into (–)-DNA. J. Biol. Chem. 297, 100889. doi: 10.1016/j.jbc.2021.100889, PMID: 34181944 PMC8321922

[B43] Choi C.-H. No J. S. Kim J.-W. Lee M. Shin H. Choi M.-M. . (2022). Complete genome sequence of monkeypox virus strain MPXV-ROK-P1–2022 isolated from the first monkeypox patient in the Republic of Korea. Microbiol. Resour. Announc. 11, e00853–e00822. doi: 10.1128/mra.00853-22, PMID: 36250860 PMC9670986

[B44] Ci Ng F. Y. Yeh S. Smit D. Ng O. T. Vasoo S. Land Curi A. L. . (2023). Monkeypox and ocular implications in humans. Ocular Surf. 27, 13–15. doi: 10.1016/j.jtos.2022.10.005, PMID: 36351509 PMC9637310

[B45] Colavita F. Mazzotta V. Rozera G. Abbate I. Carletti F. Pinnetti C. . (2023). Kinetics of viral DNA in body fluids and antibody response in patients with acute Monkeypox virus infection. IScience 26, 106102. doi: 10.1016/j.isci.2023.106102, PMID: 36748085 PMC9893533

[B46] Croft D. R. Sotir M. J. Williams C. J. Kazmierczak J. J. Wegner M. V. Rausch D. . (2007). Occupational risks during a monkeypox outbreak, Wisconsi. Emerg. Infect. Dis. 13, 1150–1157. doi: 10.3201/eid1308.061365, PMID: 17953084 PMC2828073

[B47] D’Antonio F. Pagani G. Buca D. Khalil A. (2023). Monkeypox infection in pregnancy: a systematic review and metaanalysis. Am. J. Obstet. Gynecol. MFM 5, 100747. doi: 10.1016/j.ajogmf.2022.100747, PMID: 36096413 PMC9555294

[B48] Davies D. H. McCausland M. M. Valdez C. Huynh D. Hernandez J. E. Mu Y. . (2005). Vaccinia virus H3L envelope protein is a major target of neutralizing antibodies in humans and elicits protection against lethal challenge in mice. J. Virol. 79, 11724–11733. doi: 10.1128/JVI.79.18.11724-11733.2005, PMID: 16140750 PMC1212608

[B49] Deiana M. Lavezzari D. Mori A. Accordini S. Pomari E. Piubelli C. . (2024). Exploring viral genome profile in mpox patients during the 2022 outbreak, in a north-eastern centre of Italy. Viruses 16, 726. doi: 10.3390/v16050726, PMID: 38793608 PMC11125733

[B50] Dhana A. Hamada Y. Kengne A. P. Kerkhoff A. D. Broger T. Denkinger C. M. . (2022). Diagnostic accuracy of WHO screening criteria to guide lateral-flow lipoarabinomannan testing among HIV-positive inpatients: A systematic review and individual participant data meta-analysis. J. Infect. 85, 40–48. doi: 10.1016/j.jinf.2022.05.010, PMID: 35588942 PMC10152564

[B51] Diesterbeck U. S. Gittis A. G. Garboczi D. N. Moss B. (2018). The 2.1 Å structure of protein F9 and its comparison to L1, two components of the conserved poxvirus entry-fusion complex. Sci. Rep. 8, 16807. doi: 10.1038/s41598-018-34244-7, PMID: 30429486 PMC6235832

[B52] Di Giulio D. B. Eckburg P. B. (2004). Human monkeypox: an emerging zoonosis. Lancet Infect. Dis. 4, 15–25. doi: 10.1016/S1473-3099(03)00856-9, PMID: 14720564 PMC9628772

[B53] Dong H. Xu S. Li P. Ruan W. (2025). The battle between infectious bronchitis virus and innate immunity: A mini review. Virology 603, 110321. doi: 10.1016/j.virol.2024.110321, PMID: 39644586

[B54] Duong M. T. Tebas P. Ancha B. Baron J. Chary P. Isaacs S. N. . (2024). Combination of extended antivirals with antiretrovirals for severe mpox in advanced human immunodeficiency virus infection: case series of 4 patients. Open Forum Infect. Dis. 11. doi: 10.1093/ofid/ofae110, PMID: 38486814 PMC10939438

[B55] Enserink M. (2003). U.S. Monkeypox outbreak traced to Wisconsin pet dealer. Science 300, 1639–1639. doi: 10.1126/science.300.5626.1639a, PMID: 12805511

[B56] Espada C. E. da Rocha E. L. Ricciardi-Jorge T. dos Santos A. A. Soares Z. G. Malaquias G. . (2024). ISG15/USP18/STAT2 is a molecular hub regulating IFN I-mediated control of Dengue and Zika virus replication. Front. Immunol. 15. doi: 10.3389/fimmu.2024.1331731, PMID: 38384473 PMC10879325

[B57] European Centre for Disease Prevention and Control (2024). ECDC Recommends Enhancing Preparedness as More Imported Cases of Clade I Mpox Highly Likely. Available online at: https://static.poder360.com.br/2024/08/ECDC-recommends-enhancing-preparedness-as-more-imported-cases-of-clade-I-mpox-highly-likely.pdf (Accessed September 17, 2024).

[B58] Eustaquio P. C. (2023). Epidemiologic and clinical features of mpox in adults aged> 50 years—United States, May 2022–May 2023. MMWR Morb. Mortal. Wkly. Rep. 72, 893–896. doi: 10.15585/mmwr.mm7233a3, PMID: 37590262 PMC10441827

[B59] Faye O. Pratt C. B. Faye M. Fall G. Chitty J. A. Diagne M. M. . (2018). Genomic characterisation of human monkeypox virus in Nigeria. Lancet Infect. Dis. 18, 246. doi: 10.1016/S1473-3099(18)30043-4, PMID: 29361427 PMC9628790

[B60] Finamor L. P. S. Mendes-Correa M. C. Rinkevicius M. Macedo G. Sabino E. C. Villas-Boas L. S. . (2024). Ocular manifestations of Monkeypox virus (MPXV) infection with viral persistence in ocular samples: A case series. Int. J. Infect. Dis. 146, 107071. doi: 10.1016/j.ijid.2024.107071, PMID: 38710273

[B61] Fine P. E. M. Jezek Z. Grab B. Dixon H. (1988). The transmission potential of monkeypox virus in human populations. Int. J. Epidemiol. 17, 643–650. doi: 10.1093/ije/17.3.643, PMID: 2850277

[B62] Fleischauer A. T. Kile J. C. Davidson M. Fischer M. Karem K. L. Teclaw R. . (2005). Evaluation of human-to-human transmission of monkeypox from infected patients to health care workers. Clin. Infect. Dis. 40, 689–694. doi: 10.1086/427805, PMID: 15714414

[B63] Foo C. H. Lou H. Whitbeck J. C. Ponce-de-León M. Atanasiu D. Eisenberg R. J. . (2009). Vaccinia virus L1 binds to cell surfaces and blocks virus entry independently of glycosaminoglycans. Virology 385, 368–382. doi: 10.1016/j.virol.2008.12.019, PMID: 19162289 PMC2693012

[B64] Formenty P. Muntasir M. O. Damon I. Chowdhary V. Opoka M. L. Monimart C. . (2010). Human monkeypox outbreak caused by novel virus belonging to Congo Basin Clade, Suda. Emerg. Infect. Dis. 16, 1539–1545. doi: 10.3201/eid1610.100713, PMID: 20875278 PMC3294404

[B65] Gashema P. Musafiri T. Ndahimana F. Iradukunda H. Saramba E. Nyakatswau S. T. . (2024). Mpox in East Africa: learning from COVID-19 and ebola to strengthen public health responses. Viruses 16, 1578. doi: 10.3390/v16101578, PMID: 39459912 PMC11512314

[B66] Gelderblom H. Madeley D. (2018). Rapid viral diagnosis of orthopoxviruses by electron microscopy: optional or a must? Viruses 10, 142. doi: 10.3390/v10040142, PMID: 29565285 PMC5923436

[B67] Greenwald Z. R. Bouck Z. McLean E. Mason K. Lettner B. Broad J. . (2023). Integrated supervised consumption services and hepatitis C testing and treatment among people who inject drugs in Toronto, Canada: A cross-sectional analysis. J. Viral Hepatitis 30, 160–171. doi: 10.1111/jvh.13780, PMID: 36461705

[B68] Greseth M. D. Traktman P. (2022). The life cycle of the vaccinia virus genome. Annu. Rev. Virol. 9, 239–259. doi: 10.1146/annurev-virology-091919-104752, PMID: 35584888

[B69] Grossegesse M. Stern D. Hofmann N. Surtees R. Kohl C. Michel J. . (2023). Serological methods for the detection of antibodies against monkeypox virus applicable for laboratories with different biosafety levels. J. Med. Virol. 95, e29261. doi: 10.1002/jmv.29261, PMID: 38054557

[B70] Guarner J. Johnson B. J. Paddock C. D. Shieh W.-J. Goldsmith C. S. Reynolds M. G. . (2004). Monkeypox transmission and pathogenesis in prairie dogs. Emerg. Infect. Dis. 10, 426. doi: 10.3201/eid1003.030878, PMID: 15109408 PMC3322777

[B71] Gupta A. K. Talukder M. Rosen T. Piguet V. (2023). Differential diagnosis, prevention, and treatment of mpox (Monkeypox): A review for dermatologists. Am. J. Clin. Dermatol. 24, 541–556. doi: 10.1007/s40257-023-00778-4, PMID: 37106278 PMC10136400

[B72] Hammad Q. Alalshaikh Z. M. Zeidan Z. A. Islam S. Haya A. (2024). Unusual neurological complications in a patient with monkeypox: A case report. Cureus. 16, e58479. doi: 10.7759/cureus.58479, PMID: 38765399 PMC11101155

[B73] Harapan H. Ophinni Y. Megawati D. Frediansyah A. Mamada S. S. Salampe M. . (2022). Monkeypox: A comprehensive review. Viruses 14, 2155. doi: 10.3390/v14102155, PMID: 36298710 PMC9612348

[B74] Heymann D. L. Szczeniowski M. Esteves K. (1998). Re-emergence of monkeypox in Africa: a review of the past six years. Br. Med. Bull. 54, 693–702. doi: 10.1093/oxfordjournals.bmb.a011720, PMID: 10326294

[B75] Hobbs K. J. Bayless R. Sheats M. K. (2024). A comparative review of cytokines and cytokine targeting in sepsis: from humans to horses. Cells 13, 1489. doi: 10.3390/cells13171489, PMID: 39273060 PMC11394191

[B76] Holthaus D. Vasou A. Bamford C. G. G. Andrejeva J. Paulus C. Randall R. E. . (2019). Direct antiviral activity of interferon stimulated genes is responsible for resistance to paramyxoviruses in ISG15-deficient cells. (Cold Spring Harbor, NY, USA: Cold Spring Harbor Laboratory). doi: 10.1101/2019.12.12.873919 PMC7311202 32423918

[B77] Hooper C. (1998). Poxvirus Dilemmas. New Engl. J. Med. 339, 2027–2028. doi: 10.1056/NEJM199812313392717, PMID: 9882210

[B78] Huang Y. Mu L. Wang W. (2022). Monkeypox: epidemiology, pathogenesis, treatment and prevention. Signal Transduct. Target. Ther. 7, 373. doi: 10.1038/s41392-022-01215-4, PMID: 36319633 PMC9626568

[B79] Hunt J. H. Jones J. Gebo K. Hansoti B. Traut C. C. Hamill M. M. . (2024). Discordant Performance of Mpox Serological Assays. (Rochester, NY, USA: Social Science Research Network). doi: 10.2139/ssrn.4828203 PMC11683876 39127186

[B80] Huston J. Curtis S. Egelund E. F. (2023). Brincidofovir: A novel agent for the treatment of smallpox. Ann. Pharmacother. 57, 1198–1206. doi: 10.1177/10600280231151751, PMID: 36688308

[B81] Hutin Y. J. Williams R. J. Malfait P. Pebody R. Loparev V. N. Ropp S. L. . (2001). Outbreak of human monkeypox, Democratic Republic of Congo 1996 to 1997. Emerg. Infect. Dis. 7, 434. doi: 10.3201/eid0703.017311, PMID: 11384521 PMC2631782

[B82] Ilic I. Zivanovic Macuzic I. Ilic M. (2022). Global outbreak of human monkeypox in 2022: update of epidemiology. Trop. Med. Infect. Dis. 7, 264. doi: 10.3390/tropicalmed7100264, PMID: 36288005 PMC9609983

[B83] Infezioni in Medicina (2023). Infezioni in Medicina, (Rome, Italy: Le Infezioni in Medicina) Vol. 31. doi: 10.53854/liim-3102-6, PMID: 37283638 PMC10241405

[B84] Isaacs S. N. (2022). Asymptomatic infection? Another reason to consider monkeypox a disease of public health concern. Ann. Internal Med. 175, 1485–1486. doi: 10.7326/M22-2457, PMID: 35969861

[B85] Jakobsdottir G. M. Brewer D. S. Cooper C. Green C. Wedge D. C. (2022). APOBEC3 mutational signatures are associated with extensive and diverse genomic instability across multiple tumour types. BMC Biol. 20, 117. doi: 10.1186/s12915-022-01316-0, PMID: 35597990 PMC9124393

[B86] Jamieson D. J. Jernigan D. B. Ellis J. E. Treadwell T. A. (2005). Emerging infections and pregnancy: west Nile virus, monkeypox, severe acute respiratory syndrome, and bioterrorism. Clinics Perinatol. 32, 765–776. doi: 10.1016/j.clp.2005.04.008, PMID: 16085032 PMC7119112

[B87] Jia J. (2024). Mechanism of viral immune evasion. Highlights Sci. Eng. Technol. 123, 387–393. doi: 10.54097/9vfzzf96

[B88] Jiang L. Xu A. Guan L. Tang Y. Chai G. Feng J. . (2024). A review of Mpox: Biological characteristics, epidemiology, clinical features, diagnosis, treatment, and prevention strategies. Exploration 5, 20230112. doi: 10.1002/exp.20230112, PMID: 40395760 PMC12087419

[B89] JYNNEOS . (2025). Smallpox vaccine in adult healthcare personnel at risk for mpox in the democratic republic of the Congo ( ClinicalTrials.gov). nct02977715.

[B90] Karbalaei M. Keikha M. (2022). Human monkeypox outbreak 2022 and asymptomatic human reservoirs: challenges and opportunities – correspondence. Int. J. Surg. 105, 106849. doi: 10.1016/j.ijsu.2022.106849, PMID: 36007810

[B91] Karimi A. Yarmohammadi H. Erinjeri J. P. (2024). Immune effects of intra-arterial liver-directed therapies. J. Vasc. Intervent. Radiol. 35, 178–184. doi: 10.1016/j.jvir.2023.10.019, PMID: 38272638 PMC11334421

[B92] Kasecker F. Borges A. F. de Abreu M. A. M. M. (2024). Manifestações dermatológicas da Monkeypox. Med. (Ribeirão Preto) 57, 205901. doi: 10.11606/issn.2176-7262.rmrp

[B93] Kasuga Y. Zhu B. Jang K.-J. Yoo J.-S. (2021). Innate immune sensing of coronavirus and viral evasion strategies. Exp. Mol. Med. 53, 723–736. doi: 10.1038/s12276-021-00602-1, PMID: 33953325 PMC8099713

[B94] Kataria R. Kaur S. Kaundal R. (2023). Deciphering the complete human-monkeypox virus interactome: Identifying immune responses and potential drug targets. Front. Immunol. 14, 1116988. doi: 10.3389/fimmu.2023.1116988, PMID: 37051239 PMC10083500

[B95] Khan G. Perveen N. (2024). The 2022 monkeypox outbreak 1 year on: The 5 Ws. Rev. Med. Virol. 16, c184. doi: 10.1002/rmv.2489, PMID: 37930054

[B96] Khan I. S M. Dixit T. Shinkre R. Ravindran S. Bandyopadhyay S. (2024). Differential diagnosis, prevention measures, and therapeutic interventions for enhanced monkeypox (Mpox) care. Cureus. doi: 10.7759/cureus.60724, PMID: 38903311 PMC11187445

[B97] Khattak S. Rauf M. A. Ali Y. Yousaf M. T. Liu Z. Wu D.-D. . (2023). The monkeypox diagnosis, treatments and prevention: A review. Front. Cell. Infect. Microbiol. 12. doi: 10.3389/fcimb.2022.1088471, PMID: 36814644 PMC9939471

[B98] Kikkert M. (2020). Innate immune evasion by human respiratory RNA viruses. J. Innate Immun. 12, 4–20. doi: 10.1159/000503030, PMID: 31610541 PMC6959104

[B99] Kindrachuk J. Arsenault R. Kusalik A. Kindrachuk K. N. Trost B. Napper S. . (2012). Systems kinomics demonstrates Congo Basin monkeypox virus infection selectively modulates host cell signaling responses as compared to West African monkeypox virus. Mol. Cell. Proteomics 11, M111-015701. doi: 10.1074/mcp.M111.015701, PMID: 22205724 PMC3433897

[B100] Kinganda-Lusamaki E. Amuri-Aziza A. Fernandez N. Makangara-Cigolo J.-C. Pratt C. Hasivirwe Vakaniaki E. . (2024). Clade I Mpox virus genomic diversity in the Democratic Republic of the Congo 2018 - 2024: Predominance of Zoonotic Transmission. Cell 188, 4–14.e6. doi: 10.1101/2024.08.13.24311951, PMID: 39454573

[B101] Koppe U. Jansen K. Schmidt A. J. Weber C. Schulze H. Kulis-Horn R. K. . (2024). Clinically inapparent mpox virus (MPXV) infections among clients of three anonymous Community Based Voluntary Counselling and Testing centres in Berlin, Germany 2022–2023. BMC Infect. Dis. 24, 613. doi: 10.1186/s12879-024-09510-x, PMID: 38902610 PMC11191340

[B102] Krabbe N. P. Mitzey A. M. Bhattacharya S. Razo E. R. Zeng X. Bekiares N. . (2025). Clade IIb Mpox virus (MPXV) vertical transmission and fetal demise in a pregnant rhesus macaque model. PloS One 20, e0320671. doi: 10.1371/journal.pone.0320671, PMID: 40168332 PMC11960918

[B103] Kulesh D. A. Loveless B. M. Norwood D. Garrison J. Whitehouse C. A. Hartmann C. . (2004). Monkeypox virus detection in rodents using real-time 3′-minor groove binder TaqMan^®^ assays on the Roche LightCycler. Lab. Invest. 84, 1200–1208. doi: 10.1038/labinvest.3700143, PMID: 15208646 PMC9827366

[B104] Kumar N. Acharya A. Gendelman H. E. Byrareddy S. N. (2022). The 2022 outbreak and the pathobiology of the monkeypox virus. J. Autoimmun. 131, 102855. doi: 10.1016/j.jaut.2022.102855, PMID: 35760647 PMC9534147

[B105] Laliberte J. P. Weisberg A. S. Moss B. (2011). The membrane fusion step of vaccinia virus entry is cooperatively mediated by multiple viral proteins and host cell components. PloS Pathog. 7, e1002446. doi: 10.1371/journal.ppat.1002446, PMID: 22194690 PMC3240603

[B106] Lansiaux E. Jain N. Laivacuma S. Reinis A. (2022). The virology of human monkeypox virus (hMPXV): A brief overview. Virus Res. 322, 198932. doi: 10.1016/j.virusres.2022.198932, PMID: 36165924 PMC9534104

[B107] Lee W. T. Hunt D. T. Kulas K. E. Howard K. M. Carson K. Lamson T. . (2023). Development of a novel serology assay for the detection of IgG antibodies to identify exposures to orthopoxviruses. J. Immunol. 210, 235.12–235.12. doi: 10.4049/jimmunol.210.Supp.235.12

[B108] Letafati A. Sakhavarz T. (2023). Monkeypox virus: A review. Microb. Pathogen. 176, 106027. doi: 10.1016/j.micpath.2023.106027, PMID: 36758824 PMC9907786

[B109] Li E. Gong Q. Zhang J. Guo X. Xie W. Chen D. . (2024). An mpox quadrivalent mRNA vaccine protects mice from lethal vaccinia virus challenge. Antiviral Res. 230, 105974. doi: 10.1016/j.antiviral.2024.105974, PMID: 39089331

[B110] Li C. He W.-F. Li L.-X. Chen J. Yang G.-Q. Chang H.-T. . (2022). Interferon-stimulated gene 15 knockout in mice impairs IFNα-mediated antiviral activity. Viruses 14, 1862. doi: 10.3390/v14091862, PMID: 36146669 PMC9502845

[B111] Liang H. Chen C. Liu T. Dong W. Li L. (2024). Quantitative detection of mpox antigen using time-resolved fluorescence immunochromatography. Biotechnol. Appl. Biochem. 71, 1025–1031. doi: 10.1002/bab.2594, PMID: 38689530

[B112] Lim S. Y. Jo H. J. Lee S. Y. Ahn M. Kim Y. Jeon J. . (2024). Clinical features of mpox patients in Korea: A multicenter retrospective study. J. Korean Med. Sci. 39, e19. doi: 10.3346/jkms.2024.39.e19, PMID: 38288533 PMC10825456

[B113] Lin C.-L. Chung C.-S. Heine H. G. Chang W. (2000). Vaccinia virus envelope H3L protein binds to cell surface heparan sulfate and is important for intracellular mature virion morphogenesis and virus infection *in vitro* and *in vivo*. J. Virol. 74, 3353–3365. doi: 10.1128/JVI.74.7.3353-3365.2000, PMID: 10708453 PMC111837

[B114] Lin F. Shen K. (2020). Type I interferon: From innate response to treatment for COVID-19. Pediatr. Invest. 4, 275–280. doi: 10.1002/ped4.12226, PMID: 33376955 PMC7768291

[B115] Liu H. Li C. He W. Chen J. Yang G. Chen L. . (2022). Free ISG15 inhibits Pseudorabies virus infection by positively regulating type I IFN signaling. PloS Pathog. 18, e1010921. doi: 10.1371/journal.ppat.1010921, PMID: 36315588 PMC9648840

[B116] Liu Z. Zhuan Q. Zhang L. Meng L. Fu X. Hou Y. (2022). Polystyrene microplastics induced female reproductive toxicity in mice. J. Hazard. Mater. 424, 127629. doi: 10.1016/j.jhazmat.2021.127629, PMID: 34740508

[B117] Liu B. M. Rakhmanina N. Y. Yang Z. Bukrinsky M. I. (2024). Mpox (Monkeypox) Virus and Its Co-Infection with HIV, Sexually Transmitted Infections, or Bacterial Superinfections: Double Whammy or a New Prime Culprit? Viruses 16(5), 784. doi: 10.3390/v16050784, PMID: 38793665 PMC11125633

[B118] Lourie B. Bingham P. G. Evans H. H. Foster S. O. Nakano J. H. Herrmann K. L. (1972). Human infection with monkeypox virus: laboratory investigation of six cases in West Africa. Bull. World Health Organ. 46, 633., PMID: 4340223 PMC2480791

[B119] Lucena-Neto F. D. Falcão L. F. M. Vieira-Junior A. S. Moraes E. C. S. David J. P. F. Silva C. C. . (2023). Monkeypox virus immune evasion and eye manifestation: beyond eyelid implications. Viruses 15, 2301. doi: 10.3390/v15122301, PMID: 38140542 PMC10747317

[B120] Lum K. K. Cristea I. M. (2022). Host innate immune response and viral immune evasion during alphaherpesvirus infection. Curr. Issues Mol. Biol. 42, 635–686. doi: 10.21775/cimb.042.635, PMID: 33640867 PMC8301600

[B121] MacIntyre C. R. Grulich A. E. (2022). Is Australia ready for monkeypox? Med. J. Aust. 217, 193–194. doi: 10.5694/mja2.51647, PMID: 35842889

[B122] Magnus P.v. Andersen E. K. Petersen K. B. Birch-Andersen A. (1959). A pox-like disease in cynomolgus monkeys. Acta Pathol. Microbiol. Scand. 46, 156–176. doi: 10.1111/j.1699-0463.1959.tb00328.x

[B123] Manfrini N. Notarbartolo S. Grifantini R. Pesce E. (2024). SARS-coV-2: A glance at the innate immune response elicited by infection and vaccination. Antibodies 13, 13. doi: 10.3390/antib13010013, PMID: 38390874 PMC10885122

[B124] Marennikova S. S. Šeluhina E. M. Mal’Ceva N. N. Čimiškjan K. L. Macevič G. R. (1972). Isolation and properties of the causal agent of a new variola-like disease (monkeypox) in man. Bull. World Health Organ. 46, 599., PMID: 4340219 PMC2480798

[B125] Martínez-Fernández D. E. Fernández-Quezada D. Casillas-Muñoz F. A. G. Carrillo-Ballesteros F. J. Ortega-Prieto A. M. Jimenez-Guardeño J. M. . (2023). Human Monkeypox: a comprehensive overview of epidemiology, pathogenesis, diagnosis, treatment, and prevention strategies. Pathogens 12, 947. doi: 10.3390/pathogens12070947, PMID: 37513794 PMC10384102

[B126] Masirika L. M. Kumar A. Dutt M. Ostadgavahi A. T. Hewins B. Nadine M. B. . (2024). Complete genome sequencing, annotation, and mutational profiling of the novel clade I human mpox virus, kamituga strain. J. Infect. Develop. Countries 18, 600–608. doi: 10.3855/jidc.20136, PMID: 38728644

[B127] Matho M. H. Schlossman A. Gilchuk I. M. Miller G. Mikulski Z. Hupfer M. . (2018). Structure–function characterization of three human antibodies targeting the vaccinia virus adhesion molecule D8. J. Biol. Chem. 293, 390–401. doi: 10.1074/jbc.M117.814541, PMID: 29123031 PMC5766908

[B128] Mauldin M. R. McCollum A. M. Nakazawa Y. J. Mandra A. Whitehouse E. R. Davidson W. . (2022). Exportation of monkeypox virus from the African continent. J. Infect. Dis. 225, 1367–1376. doi: 10.1093/infdis/jiaa559, PMID: 32880628 PMC9016419

[B129] Mbala P. K. Huggins J. W. Riu-Rovira T. Ahuka S. M. Mulembakani P. Rimoin A. W. . (2017). Maternal and fetal outcomes among pregnant women with human monkeypox infection in the Democratic Republic of Congo. J. Infect. Dis. 216, 824–828. doi: 10.1093/infdis/jix260, PMID: 29029147

[B130] McCollum A. M. Damon I. K. (2014). Human monkeypox. Clin. Infect. Dis. 58, 260–267. doi: 10.1093/cid/cit703, PMID: 24158414 PMC5895105

[B131] McFadden G. (2005). Poxvirus tropism. Nat. Rev. Microbiol. 3, 201–213. doi: 10.1038/nrmicro1099, PMID: 15738948 PMC4382915

[B132] Mechanisms of tecovirimat antiviral activity and poxvirus resistance. doi: 10.21203/rs.3.rs-5002222/v1., PMID: PMC11879855 39939832

[B133] Meyer H. Perrichot M. Stemmler M. Emmerich P. Schmitz H. Varaine F. . (2002). Outbreaks of disease suspected of being due to human monkeypox virus infection in the Democratic Republic of Congo in 2001. J. Clin. Microbiol. 40, 2919–2921. doi: 10.1128/JCM.40.8.2919-2921.2002, PMID: 12149352 PMC120683

[B134] Minhaj F. S. Petras J. K. Brown J. A. Mangla A. T. Russo K. Willut C. . (2022). CDC monkeypox emergency response team; CDC monkeypox emergency response team. Orthopoxvirus testing challenges for persons in populations at low risk or without known epidemiologic link to monkeypox - United State. MMWR Morb. Mortal. Wkly. Rep. 71, 1155–1158. doi: 10.15585/mmwr.mm7136e1, PMID: 36074752 PMC9470221

[B135] Mińko A. Turoń-Skrzypińska A. Rył A. Mańkowska K. Cymbaluk-Płoska A. Rotter I. (2024). The importance of the concentration of selected cytokines (IL-6, IL-10, IL-12, IL-15, TNF-α) and inflammatory markers (CRP, NLR, PLR, LMR, SII) in predicting the course of rehabilitation for patients after COVID-19 infection. Biomedicines 12, 2055. doi: 10.3390/biomedicines12092055, PMID: 39335569 PMC11429050

[B136] Mitjà O. Ogoina D. Titanji B. K. Galvan C. Muyembe J.-J. Marks M. . (2023). Monkeypox. Lancet 401, 60–74. doi: 10.1016/S0140-6736(22)02075-X, PMID: 36403582 PMC9671644

[B137] Mohapatra R. K. Singh P. K. Branda F. Mishra S. Kutikuppala L. V. S. Suvvari T. K. . (2024). Transmission dynamics, complications and mitigation strategies of the current mpox outbreak: A comprehensive review with bibliometric study. Rev. Med. Virol. 34, e2541. doi: 10.1002/rmv.2541, PMID: 38743385

[B138] Morris S. Joshi P. Soni P. Jakhmola V. Kalita K. Nainwal N. . (2023). A systematic review on human monkeypox virus disease and infection in pregnancy. J. Pure Appl. Microbiol. 17, 650–659. doi: 10.22207/JPAM.17.2.52

[B139] Moss B. (2016). Membrane fusion during poxvirus entry. Semin. Cell Dev. Biol. 60, 89–96. doi: 10.1016/j.semcdb.2016.07.015, PMID: 27423915 PMC5161597

[B140] Mpox (2022-2024). (Monkeypox) Outbreak Global Trends. Available online at: https://worldhealthorg.shinyapps.io/mpx_global/6_Genomic_epidemiology.

[B141] Mukareem Ali S. Abbasher Hussien Mohamed Ahmed K. Ahsan A. E. Mustafa Ahmed G. Fatima I. Tariq Ahmed S. . (2025). Smallpox vaccines for monkeypox: is emergency vaccination imminent? Disaster Med. Public Health Preparedness 19, e81. doi: 10.1017/dmp.2025.66, PMID: 40171842

[B142] Murray M. Peters N. Reeves M. (2018). Navigating the Host Cell Response during Entry into Sites of Latent Cytomegalovirus Infection. Pathogens 7, 30. doi: 10.3390/pathogens7010030, PMID: 29547547 PMC5874756

[B143] Nakoune E. Olliaro P. (2022). Waking up to monkeypox. BMJ 377, o1321. doi: 10.1136/bmj.o1321, PMID: 35613732

[B144] Ng S. Stephan C. Do M. Frosina D. Jungbluth A. Busam K. J. . (2025). Detecting *monkeypox* virus by immunohistochemistry. J. Cutaneous Pathol. 52, 244–249. doi: 10.1111/cup.14776, PMID: 39698761 PMC11808465

[B145] Nolen L. D. Osadebe L. Katomba J. Likofata J. Mukadi D. Monroe B. . (2015). Introduction of monkeypox into a community and household: risk factors and zoonotic reservoirs in the Democratic Republic of the Congo. Am. Soc. Trop. Med. Hyg. 93, 410–415. doi: 10.4269/ajtmh.15-0168, PMID: 26013374 PMC4530773

[B146] Number of Mpox cases per Month reported to October 2024 (Modified Source: WHO). Available online at: https://worldhealthorg.shinyapps.io/mpx_global/6_Genomic_epidemiology.

[B147] O’Neil M. J. Archer R. Danza P. Fisher R. Bagwell D. A. Younis I. . (2024). Successful distribution of tecovirimat during the peak of the mpox outbreak - Los Angeles county, June 2022-January 2023. MMWR Morb. Mortal. Wkly. Rep. 73, 546–550. doi: 10.15585/mmwr,mm7324a2, PMID: 38900699 PMC11199022

[B148] Ogoina D. Damon I. Nakoune E. (2023). Clinical review of human mpox. Clin. Microbiol. Infect. 29, 1493–1501. doi: 10.1016/j.cmi.2023.09.004, PMID: 37704017

[B149] Ogoina D. Izibewule J. H. Ogunleye A. Ederiane E. Anebonam U. Neni A. . (2019). The 2017 human monkeypox outbreak in Nigeria—report of outbreak experience and response in the Niger Delta University Teaching Hospital, Bayelsa State, Nigeria. PloS One 14, e0214229. doi: 10.1371/journal.pone.0214229, PMID: 30995249 PMC6469755

[B150] Okumura N. Morino E. Nomoto H. Yanagi M. Takahashi K. Iwasaki H. . (2024). Safety and Effectiveness of LC16m8 for Pre-Exposure Prophylaxis against mpox in a High-Risk Population: An Open-Label Randomized Trial. (Cold Spring Harbor, NY, USA: Cold Spring Harbor Laboratory). doi: 10.1101/2024.06.06.24308551 39982831

[B151] Organization W. H. (2024). *WHO Drug Information: Volume* 38 Vol. 38 ( World Health Organization).

[B152] Pauli G. Blümel J. Burger R. Drosten C. Gröner A. Gürtler L. . (2010). Orthopox Viruses: Infections in Humans. Transfus. Med. Hemother. 37, 351–364. doi: 10.1159/000322101, PMID: 21483466 PMC3048946

[B153] Ouyang M. L. Marusinec R. Bayard P. J. Edmunds M. Johnson M. Lai S. . (2024). Epidemiology of mpox cases, and tecovirimat and JYNNEOS utilization, Alameda county, California, June-October 2022. J. Public Health Manage. Pract. 30, 744–752. doi: 10.1097/PHH.0000000000002010, PMID: 39041768

[B154] Pan D. Nazareth J. Sze S. Martin C. A. Decker J. Fletcher E. . (2023). Transmission of monkeypox/mpox virus: a narrative review of environmental, viral, host, and population factors in relation to the 2022 international outbreak. J. Med. Virol. 95, e28534. doi: 10.1002/jmv.28534, PMID: 36708091 PMC10107822

[B155] Pang S. Wang M. Yuan J. Yang Z. Yu H. Zhang H. . (2024). Sensitive dual-signal ELISA based on specific phage-displayed double peptide probes with internal filtering effect to assay monkeypox virus antigen. Anal. Chem. 96, 10064–10073. doi: 10.1021/acs.analchem.4c01802, PMID: 38842443

[B156] Parker S. Buller R. M. (2013). A review of experimental and natural infections of animals with monkeypox virus between 1958 and 2012. Future Virol. 8, 129–157. doi: 10.2217/fvl.12.130, PMID: 23626656 PMC3635111

[B157] Pashazadeh Azari P. Rezaei Zadeh Rukerd M. Charostad J. Bashash D. Farsiu N. Behzadi S. . (2024). Monkeypox (Mpox) vs. Innate immune responses: Insights into evasion mechanisms and potential therapeutic strategies. Cytokine 183, 156751. doi: 10.1016/j.cyto.2024.156751, PMID: 39244831

[B158] Percentage of Symptoms in Mpox Cases Reported in 2023- Dec 2024. Available online at: https://worldhealthorg.shinyapps.io/mpx_global/.

[B159] Pérez-Martín Ó. G. Hernández-Aceituno A. Dorta-Espiñeira M. M. García-Hernández L. Larumbe-Zabala E. (2022). Atypical presentation of sexually transmitted monkeypox lesions. Infect. Dis. 54, 940–943. doi: 10.1080/23744235.2022.2121420, PMID: 36102117 PMC9527785

[B160] Piralla A. Mileto D. Rizzo A. Ferrari G. Giardina F. Gaiarsa S. . (2024). Dynamics of viral DNA shedding and culture viral DNA positivity in different clinical samples collected during the 2022 mpox outbreak in Lombardy, Italy. Travel Med. Infect. Dis. 59, 102698. doi: 10.1016/j.tmaid.2024.102698, PMID: 38556220

[B161] Pourriyahi H. Aryanian Z. Afshar Z. M. Goodarzi A. (2023). A systematic review and clinical atlas on mucocutaneous presentations of the current monkeypox outbreak: with a comprehensive approach to all dermatologic and nondermatologic aspects of the new and previous monkeypox outbreaks. J. Med. Virol. 95, e28230. doi: 10.1002/jmv.28230, PMID: 36254380

[B162] Prasetyo A. D. Hudari H. Permata M. Salin N. A. (2024). The role of tecovirimat in the management of monkeypox. Bioscientia Medicina: J. Biomed. Trans. Res. 8, 4600–4605. doi: 10.37275/bsm.v8i7.1028

[B163] Prévost J. Sloan A. Deschambault Y. Tailor N. Tierney K. Azaransky K. . (2024). Treatment efficacy of cidofovir and brincidofovir against clade II Monkeypox virus isolates. Antiviral Res. 231, 105995. doi: 10.1016/j.antiviral.2024.105995, PMID: 39243894

[B164] Probert W. S. Espinosa A. Hacker J. K. (2025). Clade I or clade II? Targeting essential viral genes to differentiate monkeypox virus clades by multiplex real-time PCR. Front. Public Health 13, 1626030. doi: 10.3389/fpubh.2025.1626030, PMID: 40823209 PMC12354367

[B165] Ramnarayan P. Mitting R. Whittaker E. Marcolin M. O’Regan C. Sinha R. . (2022). Neonatal monkeypox virus infection. New Engl. J. Med. 387, 1618–1620. doi: 10.1056/NEJMc2210828, PMID: 36223535

[B166] Rampogu S. Kim Y. Kim S.-W. Lee K. W. (2023). An overview on monkeypox virus: Pathogenesis, transmission, host interaction and therapeutics. Front. Cell. Infect. Microbiol. 13, 1076251. doi: 10.3389/fcimb.2023.1076251, PMID: 36844409 PMC9950268

[B167] Rao A. K. Schulte J. Chen T. Hughes C. M. Davidson W. Neff J. M. . (2021). Monkeypox in a traveler returning from Nigeria — Dallas, Texas, July 2021. MMWR Morb. Mortal. Wkly. Rep. 71, 509–516. doi: 10.15585/mmwr.mm7114a1, PMID: 35389974 PMC8989376

[B168] Reda A. El-Qushayri A. E. Shah J. (2023). Asymptomatic monkeypox infection: a call for greater control of infection and transmission. Lancet Microbe 4, e15–e16. doi: 10.1016/S2666-5247(22)00259-2, PMID: 36209756 PMC9536807

[B169] Reed K. D. Melski J. W. Graham M. B. Regnery R. L. Sotir M. J. Wegner M. V. . (2004). The detection of monkeypox in humans in the Western Hemisphere. New Engl. J. Med. 350, 342–350. doi: 10.1056/NEJMoa032299, PMID: 14736926

[B170] Resman Rus K. Zakotnik S. Sagadin M. Kolenc M. Skubic L. Knap N. . (2024). Review of virological methods for laboratory diagnosis and characterization of monkeypox virus (MPXV): lessons learned from the 2022 Mpox outbreak. Acta Dermatovenerologica Alpina Pannonica Adriatica 33, 23–35. doi: 10.15570/actaapa.2024.1, PMID: 38179904

[B171] Roberts K. L. Smith G. L. (2008). Vaccinia virus morphogenesis and dissemination. Trends Microbiol. 16, 472–479. doi: 10.1016/j.tim.2008.07.009, PMID: 18789694

[B172] Rothenburg S. Yang Z. Beard P. Sawyer S. L. Titanji B. Gonsalves G. . (2022). Monkeypox emergency: Urgent questions and perspectives. Cell 185, 3279–3281. doi: 10.1016/j.cell.2022.08.002, PMID: 35998628 PMC9395144

[B173] Rowe T. Fletcher A. Svoboda P. Pohl J. Hatta Y. Jasso G. . (2024). Interferon as an immunoadjuvant to enhance antibodies following influenza B infection and vaccination in ferrets. NPJ Vaccines 9, 199. doi: 10.1038/s41541-024-00973-2, PMID: 39448628 PMC11502657

[B174] Saghazadeh A. Rezaei N. (2023). Insights on Mpox virus infection immunopathogenesis. Rev. Med. Virol. 33, e2426. doi: 10.1002/rmv.2426, PMID: 36738134

[B175] Saied A. A. (2022). Is the monkeypox virus older than we think? Transboundary Emerg. Dis. 69, 2407–2408. doi: 10.1111/tbed.14651, PMID: 35789125

[B176] Saied A. A. Metwally A. A. Aiash H. (2022). Paleovirology of monkeypox virus: Egyptian animal mummies should be in focus. Lancet Microbe 3, e900. doi: 10.1016/S2666-5247(22)00298-1, PMID: 36306817 PMC9597905

[B177] Saraswat Y. Shah K. (2024). Mini review on clinical aspects of monkeypox. Curr. Pharm. Biotechnol. 25, 411–425. doi: 10.2174/1389201025666230914094444, PMID: 37711132

[B178] Savvidis C. Rizzo M. Ilias I. (2025). Mpox infection and endocrine health: bridging the knowledge gap. Medicina 61, 899. doi: 10.3390/medicina61050899, PMID: 40428857 PMC12113257

[B179] Sberna G. Rozera G. Minosse C. Bordi L. Mazzotta V. D’Abramo A. . (2024). Role of direct sexual contact in human transmission of monkeypox virus, Italy. Emerg. Infect. Dis. 30, 1829. doi: 10.3201/eid3009.240075, PMID: 39127126 PMC11346984

[B180] Schoggins J. W. Rice C. M. (2011). Interferon-stimulated genes and their antiviral effector functions. Curr. Opin. Virol. 1, 519–525. doi: 10.1016/j.coviro.2011.10.008, PMID: 22328912 PMC3274382

[B181] Schwartz D. A. Ha S. Dashraath P. Baud D. Pittman P. R. Adams Waldorf K. M. (2023). Mpox virus in pregnancy, the placenta, and newborn: An emerging poxvirus with similarities to smallpox and other orthopoxvirus agents causing maternal and fetal disease. Arch. Pathol. Lab. Med. 147, 746–757. doi: 10.5858/arpa.2022-0520-SA, PMID: 36857117

[B182] Schwartz D. A. Pittman P. R. (2023). Mpox (monkeypox) in pregnancy: Viral clade differences and their associations with varying obstetrical and fetal outcomes. Viruses 15, 1649. doi: 10.3390/v15081649, PMID: 37631992 PMC10458075

[B183] See K. C. (2022). Vaccination for monkeypox virus infection in humans: a review of key considerations. Vaccines 10, 1342. doi: 10.3390/vaccines10081342, PMID: 36016230 PMC9413102

[B184] Selb R. Werber D. Falkenhorst G. Steffen G. Lachmann R. Ruscher C. . (2022). A shift from travel-associated cases to autochthonous transmission with Berlin as epicentre of the monkeypox outbreak in Germany, May to June 2022. Eurosurveillance 27, 2200499. doi: 10.2807/1560-7917.ES.2022.27.27.2200499, PMID: 35801518 PMC9264732

[B185] Selverian C. N. Monticelli S. R. Jaleta Y. M. Lasso G. DeMouth M. E. Meola A. . (2025). Monkeypox virus infection stimulates a more robust and durable neutralizing antibody response compared to modified vaccinia virus Ankara vaccination. J. Infect. Dis. 231, 1069–1073. doi: 10.1093/infdis/jiae515, PMID: 39422181

[B186] Sha Y. Huang B. Hua C. Zhu Y. Tai W. Sun J. . (2024). A clinically used anti-human papilloma virus agent (3-hydroxyphthalic anhydride-modified bovine β-lactoglobulin) has a potential for topical application to prevent sexual transmission of monkeypox virus. MedComm 5, e677. doi: 10.1002/mco2.677, PMID: 39105195 PMC11298542

[B187] Shabil M. Khatib M. N. Ballal S. Bansal P. Tomar B. S. Ashraf A. . (2024). Effectiveness of tecovirimat in mpox cases: A systematic review of current evidence. J. Med. Virol. 96. doi: 10.1002/jmv.70122, PMID: 39707867

[B188] Sheikhi S. Shirazi F. K. H. Zamani A. Jeddi M. (2025). Impact of COVID-19 on pituitary function: a cross-sectional study in southern Iran. BMC Endocr. Disord. 25, 261. doi: 10.1186/s12902-024-01790-3, PMID: 41225399 PMC12613459

[B189] Shi M. Zhang C.-Y. Zou D.-Y. Wu J. Wu N.-H. Ni L.-Y. . (2025). Immunohistochemical analyses of skin lesions with monkeypox virus A29 and A35 antibodies: A novel insight for clinical-histopathological forms. Japanese J. Infect. Dis. 78, 55–62. doi: 10.7883/yoken.JJID.2024.145, PMID: 39477523

[B190] Singh P. Sridhar S. B. Shareef J. Talath S. Mohapatra P. Khatib M. N. . (2024). The resurgence of monkeypox: Epidemiology, clinical features, and public health implications in the post-smallpox eradication era. New Microbes New Infect. 62, 101487. doi: 10.1016/j.nmni.2024.101487, PMID: 39429728 PMC11488443

[B191] Smallpox vaccine for mpox post-exposure prophylaxis: a cluster rct (smart) ClinicalTrials.gov identifier: nct05745987. Updated May 22, 2024. Available online at: https://clinicaltrials.gov/study/NCT05745987 (Accessed August 20, 2024).

[B192] Smith G. L. Vanderplasschen A. Law M. (2002). The formation and function of extracellular enveloped vaccinia virus. J. Gen. Virol. 83, 2915–2931. doi: 10.1099/0022-1317-83-12-2915, PMID: 12466468

[B193] Srivastava S. Laxmi Sharma K. Sridhar S. B. Talath S. Shareef J. . (2024). Clade Ib: a new emerging threat in the Mpox outbreak. Front. Pharmacol. 15. doi: 10.3389/fphar.2024.1504154, PMID: 39749207 PMC11693458

[B194] Stovba L. F. Chukhralya O. V. Petrov A. A. Mel’nikov S. A. Pavel’ev D. I. Borisevich S. V. (2024). Smallpox vaccine LC16m8: production, properties, and prospects. Problems Particularly Dangerous Infect. 3, 42–50. doi: 10.21055/0370-1069-2024-3-42-50

[B195] Sui Y. Xu Q. Liu M. Zuo K. Liu X. Liu J. (2022). CRISPR-Cas12a-based detection of monkeypox virus. J. Infect. 85, 702–769. doi: 10.1016/j.jinf.2022.08.043, PMID: 36084670 PMC9628776

[B196] Suleman M. Ahmad T. Shah K. Albekairi N. A. Alshammari A. Khan A. . (2024). Exploring the natural products chemical space to abrogate the F3L-dsRNA interface of monkeypox virus to enhance the immune responses using molecular screening and free energy calculations. Front. Pharmacol. 14. doi: 10.3389/fphar.2023.1328308, PMID: 38269277 PMC10805857

[B197] Svecova D. (2024). Update on monkeypox virus infection. J. Med. Healthc. 6, 2–3. doi: 10.47363/JMHC/2024. SRC/JMHC-344.

[B198] Taha T. Y. Townsend M. B. Pohl J. Karem K. L. Damon I. K. Mbala Kingebeni P. . (2023). Design and optimization of a monkeypox virus-specific serological assay. Pathogens 12, 396. doi: 10.3390/pathogens12030396, PMID: 36986317 PMC10054672

[B199] The World Health Organization (2022). Laboratory testing or the monkeypox virus: Interim guidance. Available online at: https://www.who.int/publications/i/item/WHO-MPXlaboratory-2022.1 (Accessed January 10, 2023).

[B200] The World Health Organization (WHO) (2023). 2022 Mpox Outbreak: Global Trends. Available online at: https://worldhealthorg.shinyapps.io/mpx_global/ (Accessed January 19, 2023).

[B201] Thirunavukarasu P. Sathaiah M. Gorry M. C. O’Malley M. E. Ravindranathan R. Austin F. . (2013). A rationally designed A34R mutant oncolytic poxvirus: improved efficacy in peritoneal carcinomatosis. Mol. Ther. 21, 1024–1033. doi: 10.1038/mt.2013.27, PMID: 23439499 PMC3666626

[B202] Vakaniaki E. H. Kacita C. Kinganda-Lusamaki E. O’Toole Á. Wawina-Bokalanga T. Mukadi-Bamuleka D. . (2024). Sustained human outbreak of a new MPXV clade I lineage in eastern Democratic Republic of the Congo. Nat. Med. 30, 2791–2795. doi: 10.1038/s41591-024-03130-3, PMID: 38871006 PMC11485229

[B203] Vaughan A. Aarons E. Astbury J. Brooks T. Chand M. Flegg P. . (2020). Human-to-human transmission of monkeypox virus, United Kingdom, October 2018. Emerg. Infect. Dis. 26, 782. doi: 10.3201/eid2604.191164, PMID: 32023204 PMC7101111

[B204] Verma A. Nazli Khatib M. Datt Sharma G. Pratap Singh M. Bushi G. Ballal S. . (2024). Mpox 2024: New variant, new challenges, and the looming pandemic. Clin. Infect. Pract. 24, 100394. doi: 10.1016/j.clinpr.2024.100394

[B205] Wang H. Yin P. Zheng T. Qin L. Li S. Han P. . (2024). Rational design of a ‘two-in-one’immunogen DAM drives potent immune response against mpox virus. Nat. Immunol. 25, 307–315. doi: 10.1038/s41590-023-01715-7, PMID: 38182667

[B206] Wawina-Bokalanga T. Merritt S. Kinganda-Lusamaki E. Jansen D. Halbrook M. Pukuta-Simbu E. . (2024). Epidemiology and Phylogenomic Characterization of Distinct 2023 and 2024 Mpox outbreaks in Kinshasa, Democratic Republic of the Congo - Evidence for increasingly sustained human-to-human transmission of subclade Ia. Lancet 406, 63–75. doi: 10.1101/2024.11.15.24317404 40617661

[B207] Witwit H. Cubitt B. Khafaji R. Castro E. M. Goicoechea M. Lorenzo M. M. . (2024). Repurposing Drugs for Synergistic Combination Therapies to Counteract Monkeypox Virus Tecovirimat Resistance. (Basel, Switzerland: MDPI AG) doi: 10.20944/preprints202412.1947.v1 (Accessed January 15, 2025). PMC11769280 39861882

[B208] Witwit H. Cubitt B. Khafaji R. Castro E. M. Goicoechea M. Lorenzo M. M. . (2025). Repurposing drugs for synergistic combination therapies to counteract monkeypox virus tecovirimat resistance. Viruses 17, 92. doi: 10.3390/v17010092, PMID: 39861882 PMC11769280

[B209] World Health Organization (WHO) . (2022). WHO recommends a new name for monkeypox disease. Available online at: https://www.who.int/news/item/28-11-2022-who-recommends-new-name-for-monkeypox-disease (Accessed October 10, 2024).

[B210] World Health Organization (WHO) (2022a). Surveillance, case investigation, and contact tracing for mpox (monkeypox): interim guidance. Available online at: https://iris.who.int/handle/10665/365398 (Accessed April 19, 2024).

[B211] World Health Organization (WHO) (2022b). Monkeypox - United Kingdom of Great Britain and Northern Ireland n.d. Available online at: https://www.who.int/emergencies/disease-outbreak-news/item/2022-DON383 (Accessed February 21 2023).

[B212] World Health Organization (WHO) (2024). Director-General Declares Mpox Outbreak a Public Health Emergency of International Concern (Geneva, Switzerland: World Health Organization). Available online at: https://www.who.int/news/item/14-08-2024-who-director-general-declares-mpox-outbreak-a-public-health-emergency-of-international-concern (Accessed January 15, 2025).

[B213] World Health Organization 2022-24 (WHO) Mpox (Monkeypox) Outbreak Global Trends produced on 24 December 2024. Available online at: https://worldhealthorg.shinyapps.io/mpx_global/4_Global_situation_update (Accessed January 15, 2025).

[B214] Wright C. F. Hubbs A. E. Gunasinghe S. K. Oswald B. W. (1998). A vaccinia virus late transcription factor copurifies with a factor that binds to a viral late promoter and is complemented by extracts from uninfected HeLa cells. J. Virol. 72, 1446–1451. doi: 10.1128/JVI.72.2.1446-1451.1998, PMID: 9445047 PMC124625

[B215] Xie C. Ding H. Ding J. Xue Y. Lu S. Lv H. (2022). Preparation of highly specific monoclonal antibodies against SARS-CoV-2 nucleocapsid protein and the preliminary development of antigen detection test strips. J. Med. Virol. 94, 1633–1640. doi: 10.1002/jmv.27520, PMID: 34904253 PMC9303534

[B216] Yadav P. Devasurmutt Y. Tatu U. (2022). Phylogenomic and structural analysis of the monkeypox virus shows evolution towards increased stability. Viruses 15, 127. doi: 10.3390/v15010127, PMID: 36680170 PMC9864997

[B217] Yan K. Tang L.-K. Xiao F.-F. Zhang P. Lu C.-M. Hu L.-Y. . (2023). Monkeypox and the perinatal period: what does maternal–fetal medicine need to know? World J. Pediatr. 19, 213–223. doi: 10.1007/s12519-022-00630-5, PMID: 36378482 PMC9665008

[B218] Yang Z. (2022). Monkeypox: A potential global threat? J. Med. Virol. 94, 4034. doi: 10.1002/jmv.27884, PMID: 35614026 PMC9283296

[B219] Yi X.-M. Li M. Wang S.-Y. Wang S.-H. Zeng J.-Q. Lei Y.-L. . (2025). The conserved poxvirus membrane entry-fusion apparatus component OPG147 targets MITA/STING for immune evasion. PloS Pathog. 21, e1013198. doi: 10.1371/journal.ppat.1013198, PMID: 40498864 PMC12157864

[B220] Yon H. Shin H. Shin J. I. Shin J. U. Shin Y. H. Lee J. . (2023). Clinical manifestations of human Mpox infection: A systematic review and meta-analysis. Rev. Med. Virol. 33, e2446. doi: 10.1002/rmv.2446, PMID: 37056203

[B221] Zahmatyar M. Fazlollahi A. Motamedi A. Zolfi M. Seyedi F. Nejadghaderi S. A. . (2023). Human monkeypox: history, presentations, transmission, epidemiology, diagnosis, treatment, and prevention. Front. Med. 10. doi: 10.3389/fmed.2023.1157670, PMID: 37547598 PMC10397518

[B222] Zeng J. Li Y. Jiang L. Luo L. Wang Y. Wang H. . (2023). Mpox multi-antigen mRNA vaccine candidates by a simplified manufacturing strategy afford efficient protection against lethal orthopoxvirus challenge. Emerg. Microbes Infect. 12, 2204151. doi: 10.1080/22221751.2023.2204151, PMID: 37070521 PMC10167873

[B223] Zhang X. Liu D.-A. Qiu Y. Hu R. Chen S. Xu Y. . (2025). Recent advances in mpox epidemic: global features and vaccine prevention research. Vaccines 13, 466. doi: 10.3390/vaccines13050466, PMID: 40432078 PMC12116011

[B224] Zhou Y. Chen Z. (2023). Mpox: a review of laboratory detection techniques. Arch. Virol. 168, 221. doi: 10.1007/s00705-023-05848-w, PMID: 37543543 PMC10404179

[B225] Zhu J. Chiang C. Gack M. U. (2023). Viral evasion of the interferon response at a glance. J. Cell Sci. 136, jcs260682. doi: 10.1242/jcs.260682, PMID: 37341132 PMC10411950

[B226] Zhu L. Liu Q. Hou Y. Huang B. Zhang D. Cong Z. . (2025). MPXV infection activates cGAS-STING signaling and IFN-I treatment reduces pathogenicity of mpox in CAST/EiJ mice and rhesus macaques. Cell Rep. Med. 6, 102135. doi: 10.1016/j.xcrm.2025.102135, PMID: 40398389 PMC12147887

[B227] Zinnah M. A. Uddin M. B. Hasan T. Das S. Khatun F. Hasan M. H. . (2024). The re-emergence of mpox: old illness, modern challenges. Biomedicines 12, 1457. doi: 10.3390/biomedicines12071457, PMID: 39062032 PMC11274818

[B228] Zucker J. Hazra A. Titanji B. K. (2023). Mpox and HIV—Collision of two diseases. Curr. HIV/AIDS Rep. 20, 440–450. doi: 10.1007/s11904-023-00682-w, PMID: 37994953

